# Carbohydrate conversion in spent coffee grounds: pretreatment strategies and novel enzymatic cocktail to produce value-added saccharides and prebiotic mannooligosaccharides

**DOI:** 10.1186/s13068-024-02601-6

**Published:** 2025-01-07

**Authors:** Ali Shaikh-Ibrahim, Nicola Curci, Federica De Lise, Oriana Sacco, Mauro Di Fenza, Stefany Castaldi, Rachele Isticato, André Oliveira, José P. S. Aniceto, Carlos M. Silva, Luísa Seuanes Serafim, Kristian B. R. M. Krogh, Marco Moracci, Beatrice Cobucci-Ponzano

**Affiliations:** 1 Institute of Biosciences and BioResources, National Research Council of Italy, Via P. Castellino, 111, 80131 Naples, Italy; 2 Department of Biology, University of Naples Federico II, Via V.C. Cintia, 26, 80126 Naples, Italy; 3 Department of Plant Production and Protection, Faculty of Agriculture and Veterinary Medicine, An-Najah National University, P.O. Box 707, Nablus, Palestine; 4 NBFC, National Biodiversity Future Center, 90133 Palermo, Italy; 5 CICECO—Aveiro Institute of Materials, Department of Chemistry, University of Aveiro, 3810-193 Aveiro, Portugal; 6 Present Address: Biologiens Vej 2, 2800 Novonesis, Lyngby Denmark

**Keywords:** Glycoside hydrolase, Thermostable enzymes, Lignocellulosic biomass pretreatment, Circular bioeconomy, Bioconversion

## Abstract

**Background:**

Spent coffee grounds (SCG) are the most abundant waste byproducts generated from coffee beverage production worldwide. Typically, these grounds are seen as waste and end up in landfills. However, SCG contain valuable compounds that can be valorized and used in different applications. Notably, they are rich in carbohydrates, primarily galactomannan, arabinogalactan type II, and cellulose. Within the framework of a circular bioeconomy, the targeted degradation of these polysaccharides via a tailored cocktail of carbohydrate-active enzymes offers a promising strategy for producing high-value saccharides from coffee waste.

**Results:**

In this study, various mild pretreatments were evaluated to increase the enzyme accessibility of SCG-derived biomass, reduce lignin content, and minimize hemicellulose loss. Thermostable enzymes were selected to construct an enzymatic cocktail specifically targeting cellulose and hemicelluloses in pretreated SCGs. The approach used achieved a conversion of 52% of the polysaccharide content to oligo- and monosaccharides, producing 17.4 mg of reducing sugars and 5.1 mg of monosaccharides from 50 mg of SCG. Additionally, microwave pretreatment followed by the application of a thermostable endo β-mannanase resulted in the production of 62.3 mg of mannooligosaccharides from 500 mg of SCG. In vitro experiments demonstrated that the produced mannooligosaccharides exhibited prebiotic activity, promoting the growth and biofilm formation of five probiotic bacterial strains.

**Conclusions:**

This study highlights an effective strategy for the valorization of SCG polysaccharides through mild pretreatment and customized enzymatic cocktails in a circular bioeconomic context. The production of both monosaccharides and oligosaccharides with prebiotic activity illustrates the versatility and commercial potential of SCG as a substrate for high-value saccharides. Furthermore, the use of mild pretreatment methods and thermostable enzymes minimizes chemical inputs and energy demands, aligning with sustainable processing practices. The ability to selectively target and degrade specific polysaccharides within SCG not only enhances the yield of desirable products, but also preserves key structural components, reducing waste and promoting resource efficiency.

**Supplementary Information:**

The online version contains supplementary material available at 10.1186/s13068-024-02601-6.

## Background

Lignocellulosic waste biomasses are the most abundant feedstock and generally consist of cellulose (35–50%), hemicellulose (26–35%), and lignin (14–21%), as well as other minor components [1]. The use of these biomasses in bioprocesses to produce biobased chemicals and energy from renewable resources contributes to the reduction of environmental pollution [2]. The valorization and recovery of carbohydrates from lignocellulosic food wastes align with the principles of a circular bioeconomy and sustainable resource management. Low-carbon energy inputs, sustainable supply chains, and promising conversion technologies are needed to transform renewable bioresources into high-value biobased products, materials, and fuels [3]. However, the chemical structure of lignocellulose biomasses represents one of the major bottlenecks for their valorization. Their intricate architecture makes them recalcitrant to enzymatic and chemical biotransformation, as lignin acts as a physical barrier that hinders the enzymatic decomposition of polysaccharides. This leads to a low conversion rate of organic matter into fermentable products [4]. Therefore, further efforts should focus on exploring and optimizing pretreatment methods to effectively utilize lignocellulosic biomasses as a source of high-value products. In the bioconversion process, pretreatment of lignocellulosic biomasses is a critical step for enhancing enzyme accessibility and increasing the production of reducing sugars. Compared with harsh chemical treatment, the utilization of enzymes not only results in milder working conditions with higher selectivity, but also improves the recovery yield of fermentable sugars and reduces energy costs [5]. The enzymes responsible for polysaccharide hydrolysis in lignocellulosic biomasses are glycoside hydrolases (GHs), and multiple GH activities are necessary for the efficient saccharification of complex biomass. These enzymes are classified into different families and subfamilies, as reported in the carbohydrate active enzyme database (CAZy) [6]. This categorization not only facilitates the sharing of information about specific enzyme families, but also makes it easier and more accessible to search for specific activities.

In biotechnology, enzymes from (hyper)thermophilic microorganisms (thermozymes) have attracted considerable interest because of their ability to function under conditions where mesophilic counterparts quickly denature. In addition, their thermostability reflects broader robustness, demonstrating remarkable resistance to high concentrations of salt, organic solvents, and detergents, as well as tolerance and activity at extreme pH values [7]. These features make them ideal candidates for lignocellulosic waste biomass valorization since working at high temperatures increases the efficiency and solubility of substrates, reduces the viscosity of the matrix, and avoids the risk of microbial contamination, thereby lowering overall process costs [8]. Thus, developing thermophilic enzymatic cocktails that can operate at high temperatures with greater stability is of great biotechnological interest [9].

Spent coffee grounds (SCG) are a lignocellulosic food waste product generated during the brewing of coffee. According to FAOSTAT estimates, in 2019, approximately 10.2 million tons of coffee were produced worldwide, leading to the generation of a significant amount of SCGs, which accounts for approximately 65% of the weight of green coffee beans. Moreover, two kilograms of wet SCG are produced for every kilogram of soluble coffee produced [10]. Indicatively, global coffee consumption exceeded 177 million 60 kg bags in 2023/24 according to the International Coffee Organization [11], producing a large amount of waste. Hence, transforming coffee waste into valuable products represents an eco-friendly approach that aligns with the goals of a circular bioeconomy and Sustainable Development Goals (*The Sustainable Development Goals Report*, 2022) [12]. Owing to their high carbohydrate content, SCGs are excellent renewable resources for obtaining oligosaccharides and monosaccharides through enzymatic saccharification [13]. These compounds can be further transformed through various bioprocessing routes, including but not limited to lactic acid [2], polyhydroxyalkanoates [14]*,* and short-chain organic acids [15].

**Fig. 1:**
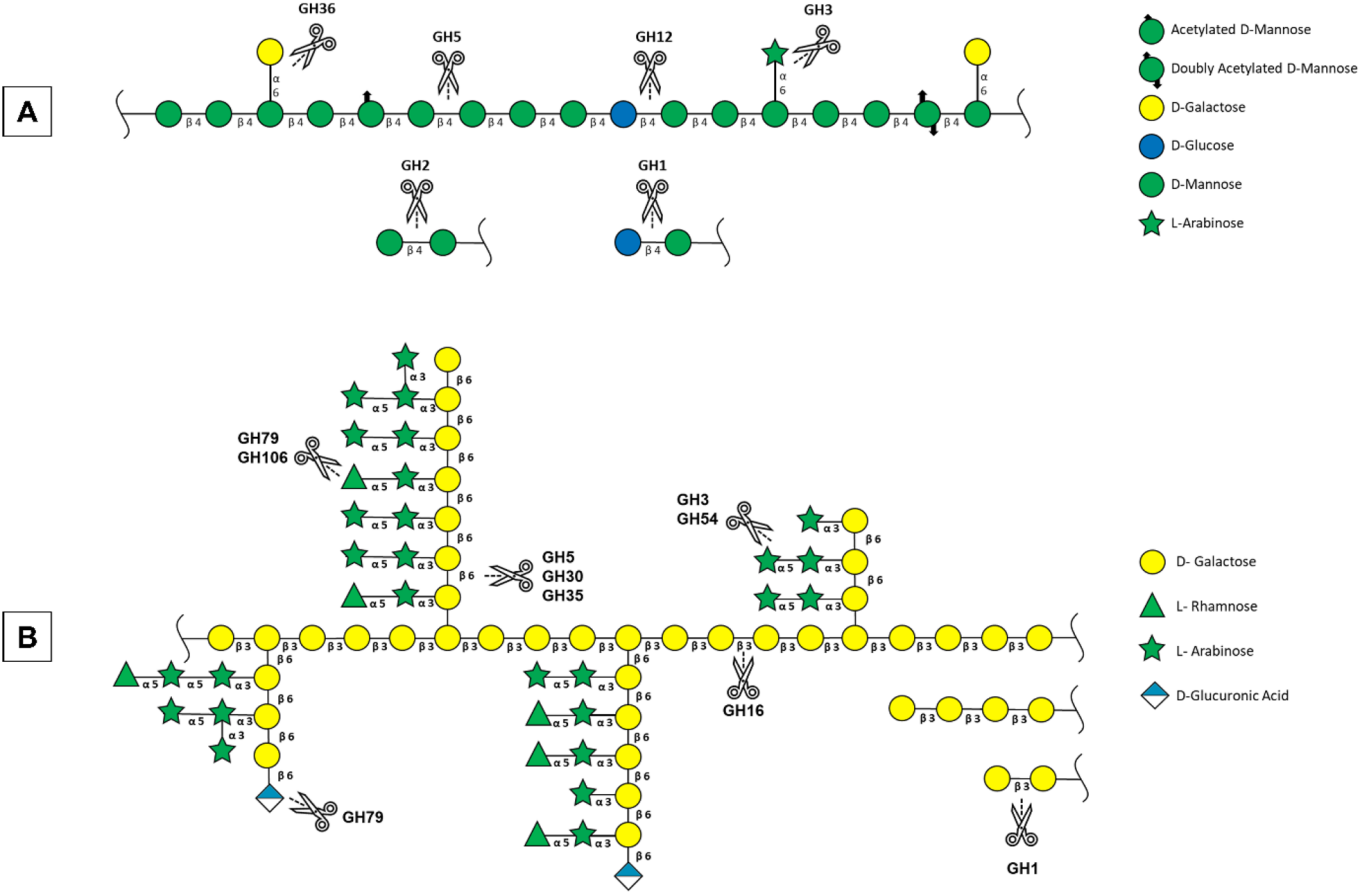
Schematic representation of galactomannan (**A**) and arabinogalactan II (**B**) structures from coffee beans and the putative glycoside hydrolases (GHs) involved in their degradation, adopted from Portillo & Arévalo [17]. Built according to the Symbol Nomenclature for Graphical Representation of Glycans (2015)

In addition to cellulose, galactomannan and arabinogalactan type II (Fig. 1A and B) are the main polysaccharides present in SCGs and can be valuable sources of value-added carbohydrates. The structure of galactomannan (Fig. 1A) consists of a linear backbone of β-(1 → 4)-D-mannose residues, which are occasionally decorated by single residues of α-(1 → 6)-D-galactose or L-arabinose, although substitutions of acetyl groups are also observed [16, 17]. The arabinogalactan II structure (Fig. 1B) is highly branched and consists of a linear backbone of β-(1 → 3)-galactose residues with long branches of β-(1 → 6) galactose oligosaccharides. These branches are usually further decorated by α-L-arabinose and α-L-rhamnose residues [18, 19].

Enzymatic cocktails for lignocellulose biomass saccharification are focused primarily on the hydrolysis of the cellulosic component. Additionally, certain pretreatment methods often result in excessive loss of hemicellulose polysaccharide content, consequently reducing the overall yield of valuable high-value products.

This study aims to explore the sustainable utilization of SCG-derived polysaccharides through the enzymatic saccharification of pretreated SCG, contributing to the production of value-added products and reducing waste in coffee brewing. We report the setup of a thermostable enzymatic cocktail by selecting specific enzymatic activities that target the cellulose and hemicellulose components of SCG. Various mild pretreatment methods have been explored to assess saccharide loss in pretreated biomass and to evaluate enzyme accessibility and effectiveness for SCG polysaccharides after pretreatment. Moreover, considering the high percentage of galactomannan in SCGs, mannooligosaccharides (MOS) were produced as value-added product using a single enzymatic activity. Their potential as prebiotics was then tested on different probiotic bacterial strains.

The manuscript addresses the need for environmentally sustainable valorization of coffee waste. The novelty of this work lies in the integration of mild pretreatment methods with a tailored enzymatic cocktail, which enhances saccharification efficiency while minimizing saccharide loss. This approach not only facilitates the production of high-value mannooligosaccharides with prebiotic potential, but also aligns with sustainable waste valorization practices. This novel combination sets this study apart from conventional approaches, paving the way for more efficient and eco-friendly coffee waste utilization.

## Methods

### Materials

SCG were collected from spent office coffee machine pods containing 100% *Coffea robusta* beans (Caffè Borbone, Italy). All chemicals, unless otherwise indicated, were purchased from Merck Chemie GmbH (Steinheim, Germany) and AppliChem (Barcelona, Spain). Monosaccharides and MOSs were purchased from Megazyme Neogen (Scotland, UK) and Biosynth (Bratislava, Slovakia).

### Spent coffee grounds preparation and pretreatment

Approximately 200 g of SCG were collected and dried in a hot air oven at 60 °C for 48 h and then stored in a cool and dry place. In all the pretreated preparations, SCG were dispersed at a fixed ratio (10% w/v). After each pretreatment, the derived SCG were filtered through Whatman grade 1 filters (11 μm, Merck) and washed with warm water until the pH was neutralized. The derived SCG were subsequently dried in a hot air oven at 60 °C for 48 h. Finally, the derived SCG were stored in a cool and dry place.

### Chemical pretreatment

SCGs acidic and alkaline pretreatments were conducted in 100 mL glass bottles. The acidic pretreatment (AC) was performed with two concentrations of H_2_SO_4_, 0.5% (AC1) and 2% w/v (AC2), both of which were subjected to the same conditions: 121 °C for 45 min. Alkaline pretreatment (AK) was performed with 0.5% (AK1) and 2% w/v (AK2) NaOH, each incubated under four conditions: 30 °C at 120 rpm with agitation for 5 h (AK1 30 °C 5 h and AK2 30 °C 5 h), 60 °C at 120 rpm with agitation for 2 h (AK1 60 °C 2 h and AK2 60 °C 2 h), 60 °C at 120 rpm for 5 h (AK1 60 °C 5 h and AK2 60 °C 5 h), and autoclaved at 121 °C and 2 bar pressure for 45 min (AK1 121 °C 45 min and AK2 121 °C 45 min).

### Physical pretreatment

Hydrothermal SCG pretreatment (HT) was performed in an autoclave (SCG 10% w/v) were placed in 100 mL glass bottles filled with distilled water at 121 °C for one h. Supercritical CO_2_ extraction (SC-CO_2_) was performed as reported by de Melo [20] with the following modifications: 20 g of dried SCG were placed in a 0.5 L extractor (0.5 L Lab unit, model Speed-SFE from Applied Separations, Inc., Allentown, PA, USA) at 300 bar and 50 °C under a constant CO_2_ flow rate of 12 g/min for 2 h. The same procedure was conducted separately by adding 10% ethanol as a cosolvent at a flow rate of 1.7 mL/min (SC-CO_2_E). Microwave pretreatment (MW) was conducted according to the method described by Binod [21] with modifications: the Microwave Digestion System “MARS 6” (CEM, USA) and Multiwave GO Plus (Anton Paar GmbH, Austria) were used indifferently for this purpose. Both systems can provide 1000 W and reach 270 °C. SCGs were suspended in distilled water (DW) at a concentration of 10% w/v and placed into microwave vessels; the samples were then subjected to microwave treatment for a fixed duration of 10 min at two different temperatures: 140 °C (MW DW1) and 170 °C (MW DW2).

### Physicochemical pretreatment

Extracted SCG derived from the SC-CO_2_ and SC-CO_2_E were pretreated with an alkaline solution using 0.5% NaOH (SC-CO_2_ AK1, SC-CO_2_E AK1) and 2.0% NaOH (SC-CO_2_ AK2, SC-CO_2_E AK2) and maintained at 60 °C with 120 rpm agitation for 2 h. Microwave-alkaline pretreatment (MW AK1) was performed using 25 mL solutions containing 10% w/v SCG in 0.5% NaOH and placed into microwave vessels. The samples were then subjected to microwave radiation for 10 min at two different temperatures, 140 °C (MW AK1 140 °C) and 170 °C (MW AK1 170 °C).

### SCGs carbohydrate and lignin composition analysis

Raw and pretreated SCG samples were subjected to monosaccharide (MS), acid-soluble lignin (ASL), and acid-insoluble lignin (AIL) analysis after two-step acid hydrolysis according to the National Renewable Energy Laboratory (NREL) protocol [22]. The MS compositions of the control and pretreated SCGs were determined via a HPAEC-PAD IC 6000 (Thermo Scientific, Sunnyvale, USA) equipped with a CarboPac PA1 (4 × 250 mm) column. The MSs were separated by isocratic elution with 12 mM NaOH at a flow rate of 0.8 mL/min for 18 min at 35 °C. A CarboPac PA200 column (3 × 250 mm) was used for MOS analysis. The analysis was conducted at 35 °C with a flow rate of 0.5 mL/min. The elution program started with isocratic elution using 40 mM NaOH for 10 min, followed by a gradient of sodium acetate (NaOAc) from 40 to 80 mM over 25 min while maintaining the NaOH concentration constant.

The ASL content in the hydrolysis liquid was determined by spectrophotometry at 205 nm after acid hydrolysis (the extinction coefficient of lignin used was 110 g/L cm). The AIL content was determined by drying the remaining solids from the two-step acid hydrolysis process at 105 ± 3 °C until a constant weight was achieved (8 h). Ash content was not considered since, in raw SCGs, it was less than 2%.

### SCG structure analysis via Fourier transform infrared spectroscopy (FT-IR)

FT-IR was performed on raw SCGs and all pretreated SCGs to analyze structural changes as a reflection of the variations in the functional groups. A Bruker Alpha ATR FT-IR spectrometer (Ettlingen, Germany) was used following the methods of Rizwan A. and Hasmukh A. [23] with minor modifications. The FT-IR spectra were recorded from 4000 to 400 cm^−1^ with 32 scans at a resolution of 4 cm^−1^ in absorbance mode. Each sample was analyzed in triplicate. The spectral data were normalized, and the means were drawn in GraphPad Prism version 8.0.2 (Boston, Massachusetts, USA).

### Enzyme selection

Thermostable GHs were selected based on the polysaccharide composition of the SCGs and their mutual compatibility in terms of pH, temperature, and thermal stability (see Supplementary material, Table S1). GH1 β-1,3-galactosidase/β-1,4-glucosidase (CelB) [24], GH36 α-1,6-galactosidase (TmGalA) [25, 26], and GH3 α-arabinosidase (XarS) [27] were already available in our laboratory. GH2 β-mannosidase (TmManA) [28] was chosen from the literature, and the synthetic gene was purchased from Twist Bioscience (San Francisco, USA). All these enzymes were expressed and purified as stated in the literature related to each enzyme cited above. Briefly, all enzymes were produced using the *E. coli* BL21(DE3) strain, except for XarS, which was produced in the BL21(DE3) LEMO strain. After purification, the enzymes achieved an average purity of 70–80%. The yields were as follows: 51 mg/L for TmManA, 22.8 mg/L for TmGalA, 8.8 mg/L for CelB, and 6.8 mg/L for XarS. Furthermore, 6 enzymes β-1,3 galactanases (Gal3) from GH16 family, 3 enzymes β-1,6 galactanases from family GH30, one β-1,6 galactanase of family GH5 (Gal6) and one exo β-1,3 galactanase from family GH43 were provided by Novonesis (Lyngby, Denmark) as purified enzymes. Endo β-1,4-mannanase (TmMan5B) and endo β-1,4-mannanase (CtMan5A) from family GH5, and a cellulase (12A) of family GH12 were purchased from Nzytech (Lisbon, Portugal), whereas a cellobiohydrolase (CBHI) of family GH7 was purchased from Megazyme Neogen (Scotland, UK).

To confirm that the enzymes were active and stable in the conditions selected for the biotransformation process (50 °C in 100 mM sodium acetate buffer, pH 5.5), they were tested on suitable substrates, in duplicate. TmManB5 and CtMan5A were tested for their activity on commercial carob galactomannan (1% w/v) (CGal) (Megazyme, UK), TmGalA was tested on 4 mM pNP-α-D-galactopyranoside, TmManA was tested on 5 mM 4NP-β-D-mannopyranoside, Gal3 and Gal6 were tested on Larch Wood Arabinogalactan II (1% w/v) (LWAG) (Carbosynth, UK), XarS was tested on 5 mM 4NP-α-L-arabinofuranoside, 12A was tested on 0.5% carboxymethyl cellulose, and CelB was tested on 5 mM 4NP-β-D-galactopyranoside. The U/mg values of all the enzymes used in the cocktail are reported in Table S2.

### Enzymatic cocktail assays

Enzymatic cocktail assays for condition selection were conducted using various SCG concentrations (10, 25, 50, and 100 mg/mL) across all the experiments, with a fixed GH concentration of 750 µg/mL in a total volume of 1 mL. The reactions were performed at 50 °C with agitation at 180 rpm for 24, 48, 72, and 96 h, terminated by boiling for 10 min and centrifuging at 12000 × *g* for 15 min at 4 °C. The sugar contents in the hydrolysate were quantified by both Somogyi–Nelson (22) and HPAEC-PAD, as described above. The enzymatic conversion rate (% w/w) was calculated for the oligosaccharides and monosaccharides released on an SCG loading dry weight basis. All the assays were performed in duplicate. After the standard conditions were selected, the pretreated SCGs (50 mg/mL) were hydrolyzed at 50 °C for 72 h in 100 mM sodium acetate buffer (pH 5.5) with 750 µg/mL GH cocktail. The samples were quantified for RS and monomeric sugar concentrations released.

### Production of mannooligosaccharides

Mannooligosaccharide production was performed in 100 mM sodium acetate buffer, pH 5.5, in a 1 or 10 mL final volume. TmMan5B (30 µg/mL) was tested on SCGs (50 mg/mL) derived from MW AK1 140 °C, SC-CO_2_E AK2, and AK2 60 °C 2 h pretreatments. The hydrolysis was carried out at 65 °C for 72 h under constant agitation (120 rpm). The mixture was then boiled for 10 min to inactivate the enzymes and centrifuged at 12000 × *g* for 15 min at 4 °C to remove the insoluble fractions; the same procedure was performed for the reaction blank without the enzyme (replaced with enzyme buffer). The hydrolysate was filtered through an Amicon Ultra30 kDa membrane (Merck, Germany) at 4500 × *g* to remove larger impurities and enzymes. The RS and MOSs in the hydrolysates were determined via the Somogyi–Nelson and HPAEC-PAD (CarboPac PA200 column (3 × 250 mm)) methods, respectively, as previously described.

### The effects of mannooligosaccharides as prebiotics

TmManB5 reaction products (TmMOS) were evaluated on five available probiotic bacterial strains, *Bacillus subtilis* NCIB3610 [29], *Bacillus velenzensis* MV4 [30], *Priestia megaterium* MV30 [30], *Lactobacillus rhamnosus* ATCC53103, and *Lactobacillus gasseri* SF118 [31]. *Bacillus* strains were grown overnight in TY broth (10 g/L tryptone, 5 g/L yeast extract, and 8 g/L NaCl) at 37 °C with shaking at 150 rpm, harvested by centrifugation at 3000 × g for 5 min and washed three times. The growth medium was composed of an M9 salt solution (6 g/L Na_2_HPO_4_ ·2H_2_O, 3 g/L KH_2_PO_4_, 0.5 g/L NaCl, 1 g/L NH_4_CL; trace elements contained 1 mg of MnCl_2_·4H_2_O, 1.7 mg of ZnCl_2_, 0.43 mg of CuCl_2_·2H_2_O, 0.6 mg of CoCl_2_·6H_2_O, 0.6 mg of Na_2_MoO_4_·2H_2_O, 0.1 mM CaCl_2_ ·2H_2_O; 2 mM MgSO_4_ ·7H_2_O). The *Bacilli* bacteria were inoculated at a final concentration of OD_600_ 0.1 in 96-well microplates (Corning^®^). The M9 minimal media was supplemented with 0.2% and 0.5% TmMOS as carbon sources (% are calculated on the total RS). M9 minimal media supplemented with 0.2% and 0.5% glucose were used as positive controls, whereas no carbon source was used as a negative control [32]. Additionally, M9 minimal media was supplemented with a reaction blank (RB) (solution obtained after SCG incubation without enzyme) using the same volume tested for glucose and/or TmMOS (0.2%/0.5%). The 96-well microplates were incubated at 37 °C with continuous shaking, and the absorbance at 600 nm was recorded every 1 h for 21 h via a Synergy™ HTX Multi-Mode Microplate Reader (BioTek, United States). *Lactobacilli* were inoculated in MRS broth (10 g/L peptone; 8 g/L beef extract; 4 g/L yeast extract; 2 g/L ammonium citrate; 3 g/L sodium citrate; 0.1 g/L MgSO_4_; 0.05 g/L MnSO_4_; 2 g/L K_2_HPO_4_) supplemented with 1% glucose, pH 6.2, and incubated at 37 °C without shaking. After overnight incubation, the bacterial cells were harvested via centrifugation at 3000 × *g* for 5 min and washed three times in MRS broth without glucose. *Lactobacilli* were inoculated in MRS broth at 0.1 OD_600_ in 96-well microplates (Corning^®^) under the same conditions described above without shaking. The experiments were performed in triplicate. A growth curve was used to calculate the generation time.

### Biofilm formation

The ability of TmMOS to influence biofilm formation was monitored using the method described in Castaldi [33] with some modifications. Briefly, each selected strain was inoculated at 0.1 OD_600_ and incubated at 37 °C for 48 h without shaking in 96-well microplates (Corning^®^) in M9 minimal media (*Bacilli*) and MRS broth (*Lactobacilli*) under the same conditions indicated in Sect. 2.8. After incubation, the wells in each plate were washed three times with phosphate-buffered saline (PBS) (80 g/L NaCl, 2 g/L KCl, 2 g/L KH_2_PO_4_, and 11.5 g/L Na_2_HPO_4_). The cells attached to the wall of each well were stained for 30 min at room temperature with 200 µL of 0.1% (w/v) crystal violet (CV) and washed again thrice with the PBS solution to remove the unbound CV. Dye attached to the wells was extracted with 200 µL of acetone/ethanol (20:80 (v/v)) followed by measuring the absorbance at 570 nm via the Synergy^™^ HTX Multi-Mode Microplate Reader. The experiment was performed in triplicate.

### Statistical analysis

Statistical analysis of enzymatic hydrolysis, probiotic bacterial strain growth, and biofilm formation was performed using GraphPad Prism 8 software. The data are expressed as the mean ± standard deviation (S.D.). Differences among groups were compared via two-way ANOVA (Tukey’s mixed model test) for enzymatic hydrolysis conditions and one-way ANOVA (Dunnett’s multiple comparisons test) for probiotic bacterial growth and biofilm formation, as indicated in the figure legends. Differences were considered statistically significant at *p* ≤ 0.05.

## Results and discussion

### Spent coffee grounds carbohydrates and lignin contents

Galactomannan, arabinogalactan II, and cellulose are the main polysaccharides present in SCGs [34]. The lignin and carbohydrate contents in SCGs could vary with coffee bean source and roasting conditions. Therefore, analyzing and evaluating the contents of carbohydrates and lignin in SCGs are crucial for effective biomass valorization. The monosaccharide analysis of the raw SCG used in this study revealed that it is rich in carbohydrates (22.3% mannose, 14.5% galactose, 12.4% glucose, and 2.8% arabinose), corresponding to more than half of the dry weight (52%, w/w) (Table 1—line 1). The lignin content was 41.5% (w/w). These results are in agreement with those reported in other studies [34–38]. Notably, the monosaccharide content reflects the composition of SCG polysaccharides: glucose is derived primarily from cellulose, mannose from galactomannan, and galactose primarily from arabinogalactan, with a smaller contribution from the side-chain decorations of galactomannan. Additionally, arabinose is a component of arabinogalactan. This analysis allows the evaluation of the effects of pretreatment and enzymatic action on the various polysaccharides in SCGs, by monitoring the monosaccharides production.

**Table 1:** Carbohydrate and lignin contents of the raw and pretreated SCG

#	Sample	Monosaccharides content (%, w/w)				Total sugars	Total lignin (%, w/w)
		Mannose	Glucose	Galactose	Arabinose		ASL + AIL
1	Raw SCG	22.3 ± 0.4	12.4 ± 0.1	14.5 ± 0.1	2.8 ± 0.0	52.0 ± 0.7	41.5 ± 1.2
2	HT	17.5 ± 0.3	10.6 ± 0.2	10.9 ± 0.1	1.8 ± 0.0	40.8 ± 0.6	41.0 ± 0.8
3	AC1	16.7 ± 0.2	16.4 ± 0.3	2.3 ± 0.4	0.4 ± 0.0	35.8 ± 0.1	61.0 ± 1.0
4	AC2	8.2 ± 0.3	18.4 ± 0.2	0.8 ± 0.2	0.0 ± 0.0	27.4 ± 0.7	69.3 ± 2.4
5	AK1 30 °C 5 h	20.4 ± 0.4	10.7 ± 0.3	10.3 ± 0.2	2.0 ± 0.0	43.4 ± 0.9	31.6 ± 0.5
6	AK2 30 °C 5 h	33.4 ± 1.9	19.8 ± 1.5	14.9 ± 1.2	2.9 ± 0.3	71.0 ± 6.9	29.3 ± 0.4
7	AK1 60 °C 2 h	21.0 ± 1.2	11.6 ± 0.7	8.8 ± 0.4	1.7 ± 0.1	43.1 ± 2.5	28.4 ± 0.5
8	AK2 60 °C 2 h	37.0 ± 0.8	22.6 ± 0.5	15.0 ± 0.3	3.0 ± 0.0	77.6 ± 1.6	23.7 ± 0.9
9	AK1 60 °C 5 h	30.4 ± 0.3	18.6 ± 0.2	13.1 ± 0.2	2.5 ± 0.0	64.6 ± 0.4	24.7 ± 0.5
10	AK2 60 °C 5 h	33.8 ± 0.9	20.9 ± 0.1	13.0 ± 0.2	2.5 ± 0.0	70.2 ± 1.0	18.4 ± 0.7
11	AK1 121 °C 45 min	27.2 ± 0.7	16.3 ± 0.1	11.9 ± 0.1	2.3 ± 0.0	57.7 ± 0.7	26.3 ± 0.9
12	AK2 121 °C 45 min	30.4 ± 0.6	20.2 ± 0.3	12.3 ± 1.2	2.3 ± 0.2	65.2 ± 2.2	13.1 ± 0.7
13	SC-CO_2_	20.2 ± 0.4	10.5 ± 0.2	12.8 ± 0.1	2.5 ± 0.1	46.0 ± 0.6	36.3 ± 0.7
14	SC-CO_2_E	15.1 ± 0.4	7.2 ± 0.2	8.9 ± 0.3	1.7 ± 0.0	32.9 ± 0.2	33.6 ± 0.1
15	SC-CO_2_ AK1	32.3 ± 1.0	17.5 ± 0.6	16.2 ± 0.5	3.0 ± 0.1	69.0 ± 2.3	27.7 ± 0.4
16	SC-CO_2_E AK1	31.6 ± 0.8	17.3 ± 0.7	16.6 ± 0.8	3.1 ± 0.0	68.6 ± 2.4	25.4 ± 0.4
17	SC-CO_2_ AK2	32.5 ± 2.4	19.1 ± 1.8	13.1 ± 1.4	2.5 ± 0.3	67.2 ± 3.8	21.8 ± 0.2
18	SC-CO_2_E AK2	32.9 ± 0.7	18.4 ± 0.1	13.3 ± 0.2	2.6 ± 0.1	67.2 ± 0.5	19.9 ± 0.5
19	MW DW 140 °C	25.5 ± 0.4	14.1 ± 0.2	15.9 ± 0.2	2.9 ± 0.0	58.4 ± 0.9	37.9 ± 0.5
20	MW DW 170 °C	22.8 ± 0.1	13.1 ± 0.3	13.0 ± 0.1	2.2 ± 0.0	51.1 ± 0.5	36.7 ± 0.3
21	MW AK1 140 °C	33.5 ± 0.2	18.4 ± 0.4	13.3 ± 0.5	2.6 ± 0.0	67.8 ± 1.2	28.6 ± 0.4
22	MW AK1 170 °C	30.7 ± 0.4	16.6 ± 0.1	11.2 ± 0.2	2.2 ± 0.0	60.7 ± 0.3	24.6 ± 1.0

The values are presented as the means ± S.D

ASL: acid-soluble lignin; AIL: acid-insoluble lignin; HT: hydrothermal pretreatment; AC1 and AC2: 0.5% and 2% H_2_SO_4_; AK1 and AK2: 0.5% and 2% NaOH; SC-CO_2_: supercritical CO_2_; SC-CO_2_E: supercritical CO_2_ with 10% ethanol; MW: microwave; DW: distilled water; w: weight

### Pretreatment effects on carbohydrate and lignin composition

The total sugar recovery and lignin content were used to evaluate the efficiency of the different pretreatments. It is worth highlighting that a pretreatment condition resulting in significant lignin removal is not necessarily better if it also causes a high loss of saccharides. To this end, the percentages of monosaccharides (MS) and lignin, calculated as the sum of acid-soluble lignin (ASL) and acid-insoluble lignin (AIL), were determined by using 300 mg of solid yields of raw biomass and biomass obtained after each pretreatment, as described in the Methods. The data from these analyses are presented in Table 1. Additional information on the solid yield, sugar recovery, and delignification percentages is reported in Table S3.

The raw biomass contained 52 and 41.5% total sugar and lignin, respectively. The hydrothermal (HT) and acid pretreatments (AC1 and AC2) proved to be inefficient for this type of biomass, both in terms of sugar recovery and lignin removal. HT allowed the recovery of 40.8% (w/w) of the total sugar content, and no significant modification of the lignin content was observed (Table 1). AC with 0.5% (AC1) and 2% (AC2) H_2_SO_4_ (Table 1—lines 3 and 4) resulted in recoveries of 35.8% and 27.4% of the total sugars, respectively. Hence, the acid concentration exerted a significant influence on sugar recovery. On the basis of the percentage of each monosaccharide, we observed that all of them decreased except for glucose, yielding higher recovery even compared with that of the raw SCGs, as also observed in previous studies [39]. These results may suggest that cellulose is more resistant to acidic treatments, which primarily affects the hemicellulose content of the biomass. Arabinogalactan appeared to be the most sensitive to this pretreatment, as the arabinose and galactose percentages decreased significantly. Moreover, after acidic pretreatment, the lignin content was very high (61.0% and 69.3% (w/w) for AC1 and AC2, respectively), highlighting the lower delignification power on this biomass.

On the other hand, alkaline pretreatment (AK) demonstrated greater effectiveness, achieving a favorable recovery of sugars versus lignin. Indeed, this pretreatment is considered one of the most useful methods with numerous advantages, including efficient delignification, minimal impact on hemicellulose, and mild reaction conditions. Additionally, many of the reagents used in alkaline pretreatment can be recovered and reused [40]. In this study, several AK conditions (Table 1, lines 5 to 12) were evaluated. The pretreatments were performed using low NaOH concentrations (0.5% and 2% in AK1 and AK2, respectively), different temperatures (30, 60, and 120 °C), and different incubation times. After pretreatment, the highest total sugar recovery (77.6%) was obtained from the AK2 60 °C 2 h sample (line 8), whereas the lowest lignin residue was detected from the AK2 121 °C 45 min (line 12) (13.1%). Notably, according to the monosaccharide composition, under all AK conditions, the hemicellulose content was preserved.

Positive outcomes have also been observed with supercritical CO_2_ (SC-CO_2_) extraction and microwave pretreatment (MW), especially when combined with low concentrations of NaOH. Under all the conditions, no significant loss of hemicellulose was observed. SC-CO_2_ extraction can efficiently remove extractives, such as oils and lipids, from biomass [41] and potentially improve the effectiveness of subsequent steps. In this study, sugar recoveries of 46.0% in SC-CO_2_ and 32.9% in SC-CO_2_E were obtained (Table 1, lines 13 and 14). In the presence of 0.5% (Table 1, lines 15–16) and 2% NaOH (lines 17–18), an improvement in overall delignification was observed, along with an increase in total sugar recovery relative to dry weight. In fact, the total sugar percentages ranged from 67.2% to 69.0%. Concurrently, a reduction in lignin content was observed, with values ranging from 36.3% to 19.9% (Table 1, lines 13 to 18).

Microwave pretreatment (MW) is often considered among the best pretreatments for high sugar recovery [42]. This work explored four different MW conditions, as reported in the Methods section, and the results are summarized in Table 1 (from lines 19 to 22). The highest sugar recovery was achieved with a combination of 140 °C and 0.5% NaOH for 10 min (Table 1, line 21), yielding approximately 67.8% sugar recovery.

In summary, pretreatments applied to SCGs under alkaline conditions appear to enhance the overall process, especially when combined with MW and SC-CO₂. These conditions enable efficient lignin removal and sugar recovery while preserving most of the hemicellulose content. Conversely, while AC was not effective in lignin removal or sugar retention, the loss of hemicellulose and retention of cellulose could serve as promising starting points for glucose production in fermentation processes.

### SCG characterization before and after pretreatment was performed via Fourier transform infrared spectroscopy

To investigate the structural changes in lignin, cellulose, and hemicellulose after pretreatment, the samples were analyzed via FT-IR spectroscopy. The average transmission FT-IR spectra of the pretreated and untreated SCG samples showed bands typical of lignocellulosic material in all the spectra (see Supplementary material, Fig. S1). However, a comparative evaluation revealed clear differences in functional groups among pretreated SCG samples, mainly in terms of peak intensity and shifting.

The FT-IR spectra of the pretreated SCG samples confirmed changes in the structure and content of lignin and carbohydrates. In general, peaks that are characteristic of lignin, namely, those at 1372 cm⁻1, 1458 cm⁻1, and 1514 cm⁻1 [43], disappeared, whereas the peak at 1640 cm⁻1 shifted and broadened in the case of the SC-CO_2_E AK2 60 ºC 2 h pretreatment. In the case of AK2 60 ºC 2 h pretreatment, the peak at 1372 cm⁻1 presented greater absorbance, whereas those at both 1458 cm⁻1 and 1514 cm⁻1 presented lower absorbance, and the peak at 1640 cm⁻1 shifted. In MW-pretreated SCGs, the vibration intensity of all peaks increased, with peak changes and shifts in the region of lignin (1422–1,636 cm⁻1) compared to raw SCGs. This might be explained by the changes in the porosity of the structure and bond breakage with less removal of lignin. Regarding carbohydrates, the hemicellulose regions at 1740 cm⁻1 were not altered in all the conditions except SC-CO_2_ or AC pretreatments. Similar findings have also been reported elsewhere [44, 45].

### Enzymatic cocktail setup and hydrolysis condition selection

To hydrolyze SCG polysaccharides, various thermostable GH activities were identified to set up hydrolysis conditions and develop enzymatic cocktails (Table S1). For the saccharification of galactomannan (Fig. 1A), two commercially available endo β-mannanases (GH5) with proven activity toward this polysaccharide were chosen. Additionally, we employed two in-house exo-enzymes, each targeting α-linked mannose and galactose residues (GH2 and 36, respectively). For the hydrolysis of arabinogalactan II (Fig. 1B), six endo β-1,3-galactanases (GH16), four endo β-1,6-galactanases (GH5 and 30), and two exo β-galactanases (GH43 and GH1) were tested. Additionally, an α-arabinosidase (GH3) was selected to remove arabinose residues.

For cellulose hydrolysis, we selected commercial cellulase (GH12), cellobiohydrolase (GH7), and in-house β-1,4-glucosidase (GH1). This latter enzyme could also act on β-linked galactose residues in arabinogalactan II, potentially reducing the need for additional enzymes in the cocktail.

Each of the above-selected enzymes (Table S1), according to their activity, were individually tested, as well as in combination, on galactomannan (Fig. S2), arabinogalactan II (Fig. S3) and carboxy-methyl-cellulose substrates to identify the best enzyme combination to test the polysaccharide content of SCG and determine the hydrolysis reaction parameters. Taking into account the optimal pH and temperature conditions for hydrolysis specific to each enzyme, the conditions selected were 100 mM sodium acetate buffer, pH 5.5, at 50 °C. On the basis of their activity on specific substrates, long-term stability, and potential synergy, the preliminary analysis identified the following enzymes to construct the enzymatic cocktail (see Supplementary material, Table S2): TmManB5, TmManA, and TmGalA for galactomannan degradation; Gal3D, Gal6D, CelB, and XarS for arabinogalactan II; and 12A, CBHI, and CelB for cellulose.

**Fig. 2:**
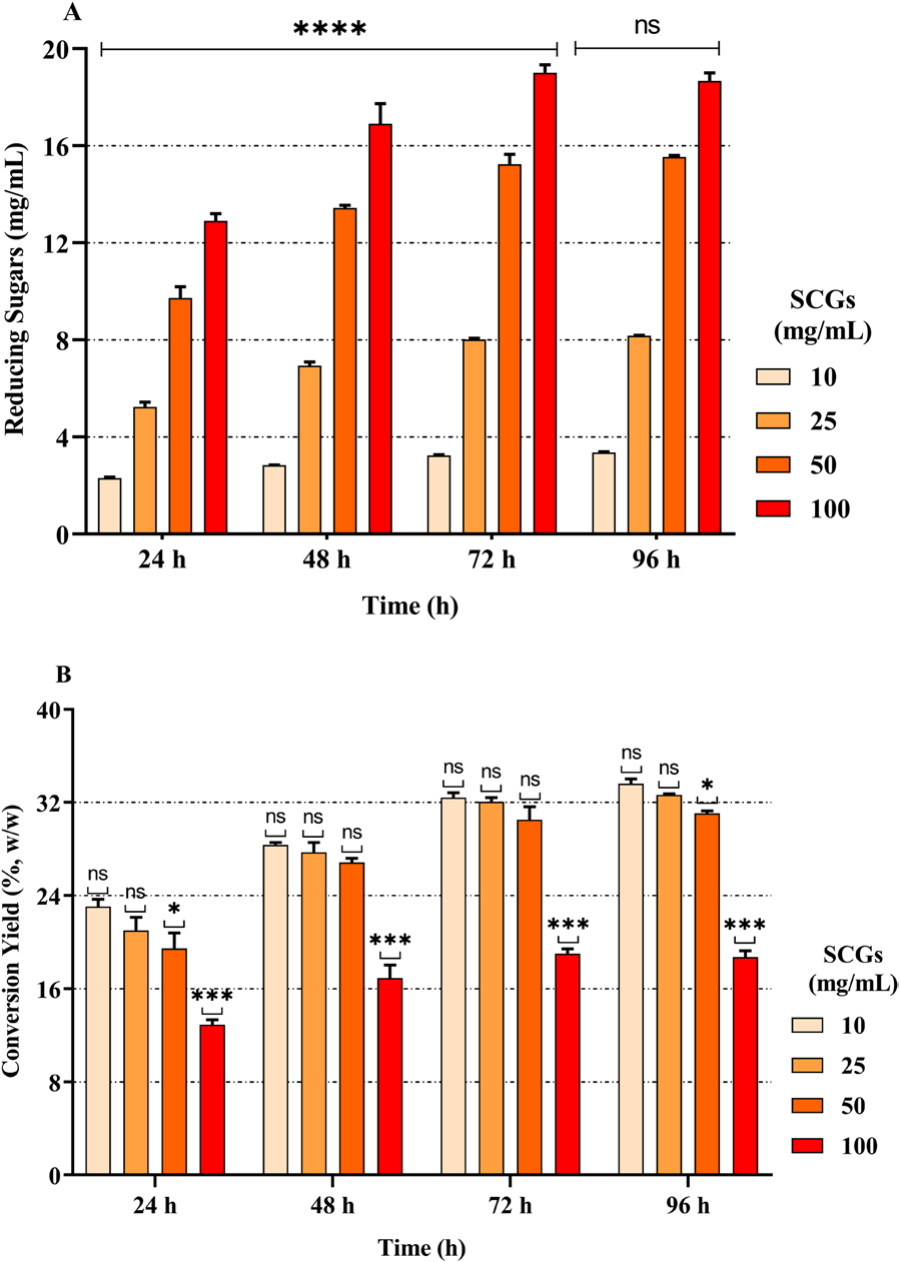
Reducing sugar recovery (**A**) and overall conversion yield (**B**) after enzymatic cocktail hydrolysis of AK2 60 °C 2 h pretreated SCG sample with different SCG concentrations (10, 25, 50, and 100 mg/mL; dry weight) for different durations (24, 48, 72, and 96 h). The values are presented as the means ± S.D.s (error bars). ****samples considered statistically significant compared with the control at *p* ≤ 0.0001; ***samples considered statistically significant compared with the control at *p* ≤ 0.001; **samples considered statistically significant compared with the mixed model at *p* ≤ 0.01; ns: not significant

To evaluate the efficiency of the enzymatic cocktail over time, saccharification tests were conducted on alkaline-pretreated SCG biomass AK2 at 60 °C for 2 h (line 8 in Table 1), which was chosen for its high total sugar recovery and low lignin content. Different concentrations of the pretreated biomass (10, 25, 50, and 100 mg/mL) were incubated with 0.75 mg/mL of the enzymatic cocktail (enzymatic units used are reported in Table S2), and the reactions were performed over 4 different time intervals, 24, 48, 72, and 96 h. The hydrolysis of SCG polysaccharides was monitored by measuring the release of RS, and the results are reported in Fig. 2A. The highest RS concentration reached approximately 19 mg/mL after 72 h by using 100 mg/mL (w/v) of pretreated biomass. Incubations beyond this time did not significantly increase the release of RS. This may be due to various factors, such as enzyme stability or the accumulation of products that may inhibit enzymes. Therefore, 3 days of incubation was chosen as the standard reaction time. After 72 h, increasing the amount of pretreated SCG to 50 mg/mL had no significant effect on the conversion yield (Fig. 2B), as the use of 100 mg/mL biomass significantly reduced the yield. Based on these observations, since 50 mg/ml pretreated SCG allowed the production of a significant amount of reducing sugars with a high conversion yield after 72 h, these reaction conditions were selected for the subsequent experiments.

### Reduced sugar and monosaccharide yields of SCGs derived from all pretreatments

The enzymatic cocktail was tested on all the pretreated SCGs. For each sample, the RS, conversion yields, and monosaccharides were identified and quantified, as summarized in Table 2. According to the results, depending on the type of pretreatment used, the recovery of carbohydrates after the enzymatic hydrolysis of SCG biomasses differs considerably in terms of efficiency and monosaccharide composition.

**Table 2:** Reducing sugars yield and the monosaccharides content from the raw and pretreated SCG after enzymatic cocktail hydrolysis

#	Sample	Total RS (mg/mL)	Conversion (%, w/w)	Mannose (%, w/w)	Glucose (%, w/w)	Galactose (%, w/w)	Arabinose (%, w/w)	Total MS (%, w/w)
1	Raw SCG	2.83 ± 0.25	5.67	1.23 ± 0.09	0.58 ± 0.07	0.38 ± 0.02	0.04 ± 0.01	2.23 ± 0.19
2	HT	3.16 ± 0.25	6.31	0.65 ± 0.02	0.51 ± 0.08	0.36 ± 0.04	0.01 ± 0.01	1.53 ± 0.09
3	AC1	4.34 ± 0.43	8.67	1.50 ± 0.10	4.05 ± 0.24	0.21 ± 0.05	0.23 ± 0.07	5.99 ± 0.37
4	AC2	6.57 ± 0.54	13.14	1.11 ± 0.04	7.19 ± 0.84	ND	ND	8.30 ± 0.80
5	AK1 30 °C 5 h	5.72 ± 0.24	11.44	1.67 ± 0.08	0.95 ± 0.06	1.22 ± 0.09	0.52 ± 0.06	4.36 ± 0.05
6	AK2 30 °C 5 h	7.65 ± 0.21	15.31	2.08 ± 0.09	1.34 ± 0.08	1.17 ± 0.07	0.46 ± 0.08	5.05 ± 0.03
7	AK1 60 °C 2 h	8.92 ± 0.42	17.85	2.90 ± 0.17	1.67 ± 0.08	1.20 ± 0.15	0.45 ± 0.13	6.22 ± 0.28
8	AK2 60 °C 2 h	15.24 ± 0.58	30.48	5.09 ± 0.29	3.43 ± 0.06	1.43 ± 0.03	ND	9.95 ± 0.37
9	AK1 60 °C 5 h	9.67 ± 0.44	19.34	3.96 ± 0.31	3.28 ± 0.13	1.12 ± 0.05	0.16 ± 0.05	8.52 ± 0.17
10	AK2 60 °C 5 h	13.31 ± 0.94	26.63	2.82 ± 0.30	3.67 ± 0.08	1.31 ± 0.04	0.28 ± 0.06	8.08 ± 0.48
11	AK1 121 °C 45	11.90 ± 0.83	23.83	2.88 ± 0.19	1.51 ± 0.09	0.93 ± 0.09	ND	5.32 ± 0.19
12	AK2 121 °C 45	14.09 ± 0.33	28.18	3.08 ± 0.15	1.53 ± 0.02	0.89 ± 0.05	ND	5.50 ± 0.08
13	SC-CO_2_	2.20 ± 0.05	4.41	0.54 ± 0.01	0.14 ± 0.03	0.12 ± 0.01	ND	0.80 ± 0.05
14	SC-CO_2_E	2.66 ± 0.09	5.33	0.55 ± 0.02	0.14 ± 0.00	0.14 ± 0.00	ND	0.83 ± 0.03
15	SC-CO_2_ AK1	9.91 ± 0.51	19.82	2.22 ± 0.09	0.90 ± 0.03	0.84 ± 0.07	ND	3.96 ± 0.19
16	SC-CO_2_E AK1	12.03 ± 0.18	24.07	3.24 ± 0.09	1.14 ± 0.04	1.02 ± 0.03	ND	5.40 ± 0.17
17	SC-CO_2_ AK2	11.40 ± 0.25	22.85	3.64 ± 0.28	0.80 ± 0.01	0.74 ± 0.26	ND	5.18 ± 0.55
18	SC-CO_2_E AK2	14.90 ± 0.62	29.81	6.25 ± 0.42	0.92 ± 0.04	0.24 ± 0.03	ND	7.41 ± 0.41
19	MW DW 140 °C	2.49 ± 0.11	4.98	0.82 ± 0.04	0.16 ± 0.01	0.21 ± 0.02	ND	1.19 ± 0.07
20	MW DW 170 °C	4.19 ± 0.04	8.39	1.23 ± 0.05	0.45 ± 0.05	0.59 ± 0.02	ND	2.27 ± 0.02
21	MW AK1 140 °C	17.40 ± 0.71	34.95	3.49 ± 0.22	3.29 ± 0.24	2.77 ± 0.10	0.74 ± 0.18	10.29 ± 1.17
22	MW AK1 170 °C	14.70 ± 0.63	29.47	2.12 ± 0.25	2.20 ± 0.27	2.13 ± 0.07	0.74 ± 0.12	7.19 ± 0.02

Values are presented as means ± S.D

RS: reducing sugars, w: weight, MS: monosaccharides, ND: not detected, HT: hydrothermal pretreatment, AC1, and AC2: 0.5%, and 2% H_2_SO_4_, AK1, and AK2: 0.5%, and 2% NaOH, SC-CO_2_: supercritical CO_2_, SC-CO_2_E: supercritical CO_2_ with 10% ethanol, MW: microwave, DW: distilled water

The enzymatic hydrolysis was more efficient for samples derived from AK alone or in combination with SC-CO_2_ and MW pretreatments, as reported in Table 2 (lines 6 to 12, 15 to 18, 21, and 22). Under these conditions, the RS yields ranged from 11.44% to 34.95%. Among them, a conversion of approximately 30% was obtained using SCGs derived from three different pretreatments: MW AK1 140 °C (35%), AK2 60 °C 2 h (30%), SC-CO_2_E AK2 (30%) (Table 2). These results agree with what was observed in the analysis of the SCG after the pretreatments (Table 1). Indeed, under these conditions, the best balance between total sugar recovery and lignin loss was achieved, highlighting the importance of setting a suitable pretreatment strategy to have biomass accessible for subsequent enzymatic hydrolysis. The enzymatic hydrolysis of MW AK1 140 °C was the most efficient, with a conversion yield of 35% (w/w), corresponding to 17.40 mg/mL RS (Table 2, line 21). These results are comparable to the highest RS yields reported to date (32%, w/w) reported in the work of Jomnonkhaow et al. [35], in which was used 50 mg/mL alkaline-pretreated SCG (4% NaOH at 60 °C for 1 h) and hydrolyzed with the commercial enzymatic cocktail Cellic^®^ CTec2 cellulase (Novozymes, Denmark) for 72 h. However, a higher concentration of NaOH was employed to pretreat the SCG, and the enzymatic cocktail used mostly targeted the cellulose fraction only. These findings suggest that the cellulose-degrading enzymes in the cocktail developed in this work are less efficient than those present in commercial cocktails. Therefore, this observation can serve as an excellent starting point for improving the enzymatic cocktail. On the other hand, we observed compensation by hemicellulose-degrading enzymes, achieving a high conversion yield comparable to that of a commercial cocktail. This highlights the importance of both preserving hemicelluloses and selecting and using enzymes that contribute to their degradation. The broad exploration of various pretreatment methods and the specific enzymatic activity of the cocktail used in this work provides an overview of the potential outcomes achievable by modulating both the pretreatment conditions and enzymatic activities, depending on the starting biomass or desired product. Indeed, looking at the different monosaccharides obtained after the enzymatic hydrolysis of pretreated SCG, it is possible to develop a strategy based on the desired end-product. For example, the action of the enzymatic cocktail on the SC-CO_2_E AK2 biomass (line 18 in Table 2) produced 14.90 mg/mL RS, with a high mannose yield (6.25% mannose out of 7.4% (w/w) total monosaccharides). Conversely, the enzymatic action on AC2 (line 4 in Table 2) converted approximately 40% of the total glucose, achieving a yield of 7.2% out of 18.4% (line 4 in Table 1), suggesting that AC2 is a good pretreatment strategy for glucose extraction from SCGs. Moreover, the highest recovery of galactose and arabinose was obtained when MW-pretreated biomass was used (Table 2). To the best of our knowledge, this is the first in-depth analysis of sugar recovery after the enzymatic hydrolysis of differently pretreated SCGs via a customized thermostable enzymatic cocktail.

### Production of mannooligosaccharides

Taking advantage of the enzyme selectivity and the results obtained, the efficiency of TmMan5B (GH5) in producing MOS from SCGs was evaluated. Coffee-derived oligosaccharides have several advantages in many biological processes, including anticancer, anti-inflammatory, gastrointestinal, and immunomodulatory functions [46]. In particular, MOS derived from SCG mannan have been used as active prebiotic ingredients and have been approved as foods for specific health uses [47]. For this purpose, SC-CO_2_E AK2 and AK2 60 °C 2 h were selected as suitable biomasses because of their high mannose recoveries after enzymatic hydrolysis, mirroring the content and accessibility of galactomannan after pretreatment. In addition, considering the low use of chemicals, the short pretreatment time, and the efficiency of enzymatic hydrolysis, MW AK1 140 °C was also evaluated for MOS production. Using 0.6 mg of TmMan5B enzyme per gram of the three selected pretreated SCGs resulted in nearly the same amount of RS being released as well as comparable amounts of mannobiose (M2) and mannotriose (M3) (see supplementary material Table S4 and Fig. S4). The MW AK1 140 °C sample was chosen for scaling up the biotransformation to 10 mL; 0.5 g of SCGs and 30 µg/mL of TmMan5B were used, and the enzyme-SCG ratio was maintained at 0.6 mg per gram. Under these conditions, 2.8 mg/mL and 0.9 mg/mL M2 and M3, respectively, were obtained, for a total of 6.23 mg/mL RS (Table S4). This result is similar to that obtained by Magengelele et al. [32], who reported M2 and M3 oligosaccharides (1.04 and 1.20 mg/mL, respectively) for a total of 1.8 mg/mL RS by using 0.25 mg of a mannanase from *Bacillus* sp. per gram of SCGs.

These results confirm the possibility of producing mannooligosaccharides with high potential commercial value from waste biomass via enzymatic hydrolysis using a single enzyme. Additionally, the enzymatic process generates fewer inhibitors and side products, simplifying purification, which can be achieved through various methods when needed [48].

### MOS effect on probiotic bacteria

**Fig. 3:**
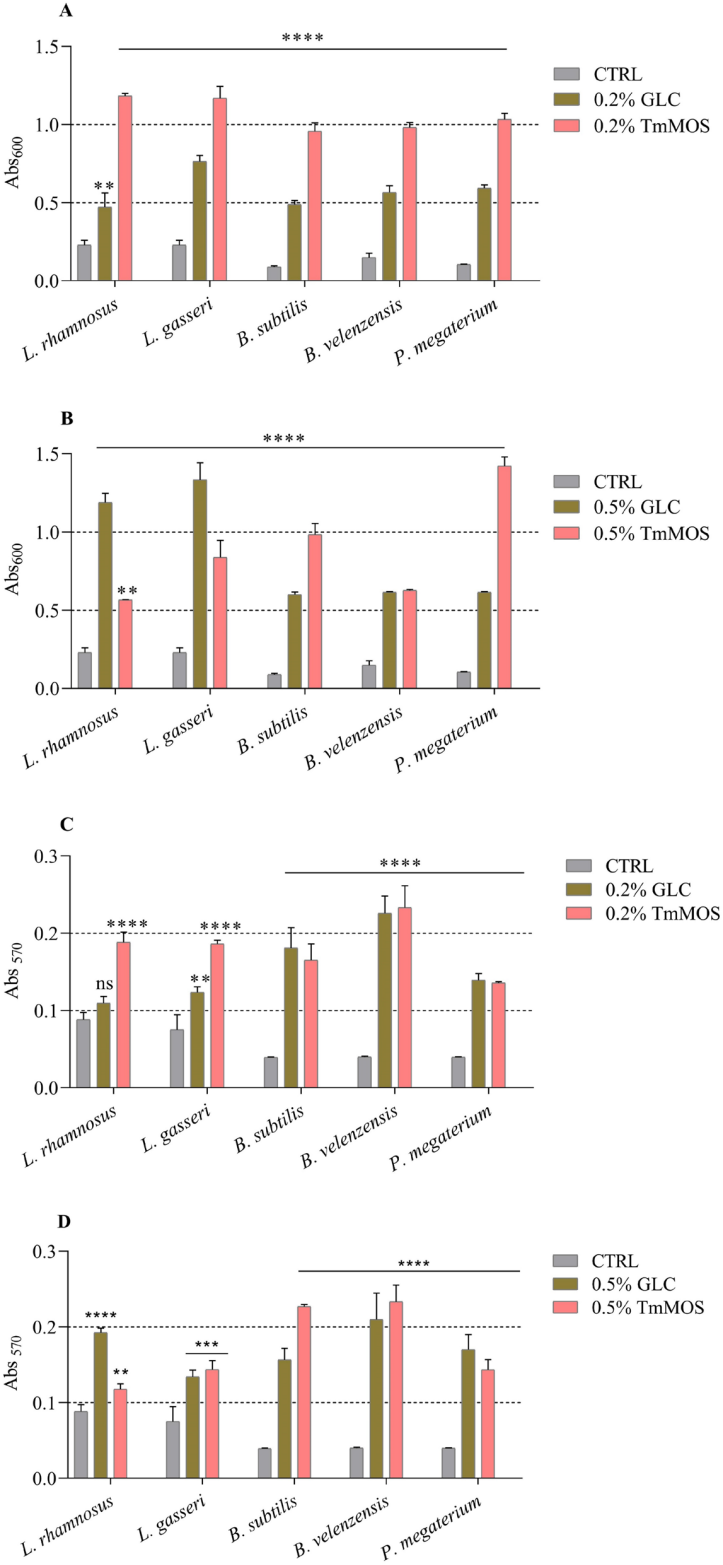
Effect of TmMOS on the growth and biofilm formation of probiotic bacteria. (**A**) Maximum Abs_600_ with 0.2% glucose/TmMOS, (**B)** maximum Abs_600_ with 0.5% glucose/TmMOS, (**C**) biofilm formation with 0.2% glucose/TmMOS, and (**D**) biofilm formation with 0.5% glucose/TmMOS. One-way ANOVA (Dunnett’s multiple comparisons test): all bacterial strains were compared with the respective control (CTRL—without a carbon source). The values are presented as the means ± S.D.s (error bars). ****samples considered statistically significant compared with the control with *p* ≤ 0.0001; ***samples considered statistically significant compared with the control with *p* ≤ 0.001; **samples considered statistically significant compared with the control with *p* ≤ 0.01. CTRL: control; GLC: glucose; ns: not significant

The prebiotic effects of MOS produced from pretreated SCG (TmMOS) on the growth of five intestinal bacteria with probiotic properties were analyzed (Fig. 3). When the growth media for *Bacilli* and *Lactobacilli* was supplemented with 0.2% TmMOS or Glc as the sole carbon source, all the analyzed strains exhibited improved growth in the presence of TmMOS (Fig. 3A). Increasing the percentage of Glc and TmMOS to 0.5% confirmed this trend for *B. subtilis* NCIB3610 and *P. megaterium* MV30; however, it was reversed for *Lactobacillus* strains, which grew better in glucose than in TmMOS.

The growth rate reduction at the increase of TmMos in *Lactobacillus* could be due to osmotic stress caused by excessive substrate concentrations, metabolic byproducts that accumulate to toxic levels, or the inability of the bacterial strain to efficiently metabolize excess oligosaccharides due to saturation of transport systems or enzymatic pathways [49]. No differences were instead recorded for *B. velenzensis* MV4 (Fig. 3B). These results confirm the positive effect of TmMOS, which, in many cases, is the preferred carbon source for gut-beneficial bacteria. These results align with those of a recent study reported by Magengelele [32], in which MOS promoted the growth and viability of *L. bulgaricus*, *S. thermophilus* and *B. subtilis* after incubation with MOS obtained from SCG hydrolysis. These results were further supported by the generation time of the beneficial bacteria tested, which doubled faster in the presence of TmMOS than in the presence of Glc (Table S5 and Fig. S5). This effect was primarily observed with the probiotic bacteria *L. gasseri* (SF1183) and *P. megaterium* (MV30). At the lowest tested concentration of TmMOS (0.2%), both exhibited maximum absorbances exceeding 1 OD600 nm (Fig. 3A), along with increases in the generation times of 25% and 75%, respectively (Table S5 and Fig. S5). Additionally, the impact of TmMOS on biofilm formation by the five bacterial strains was examined. Biofilm formation is a beneficial characteristic of probiotic bacteria, as it can extend their residence time in the intestine and inhibit colonization by enteropathogens [50]. Biofilm development is influenced by various factors, including bacterial species and strains, pH, cell surface characteristics, and culture conditions [50]. Fructo- and galacto-oligosaccharides, commonly studied in probiotics like *Lactobacillus* and *Bifidobacterium*, enhance biofilm formation by serving as substrates for bacterial growth, modifying carbohydrate-biofilm structure, interacting with bacterial surface receptors or signaling molecules such as c-di-GMP, which controls the switch between planktonic and biofilm states [50, 51]. As illustrated in Fig. 3C and D, compared with Glc, TmMOS enhanced the biofilm-forming capacity of the tested probiotic bacteria, with comparable or improved biofilm formation observed in the presence of both 0.2% and 0.5% TmMOS. Specifically, the lowest concentration of TmMOS (0.2%) significantly enhanced biofilm formation in all *Lactobacillus* strains, probably due to the growth rate increase observed in Fig. 3A. The optimal result for *B. subtilis* was instead achieved with 0.5% TmMOS.

These results are consistent with those of previous studies confirming the effectiveness of MOSs on other beneficial bacterial strains that have not been tested previously and supporting their production and use for health benefits [32, 52, 53]. Further experiments, including comparative transcriptomics, proteomics, or targeted gene knockouts and overexpression studies, could identify key regulatory elements or pathways involved in biofilm formation influenced by TmMOS.

### Mass balance evaluation

**Fig. 4:**
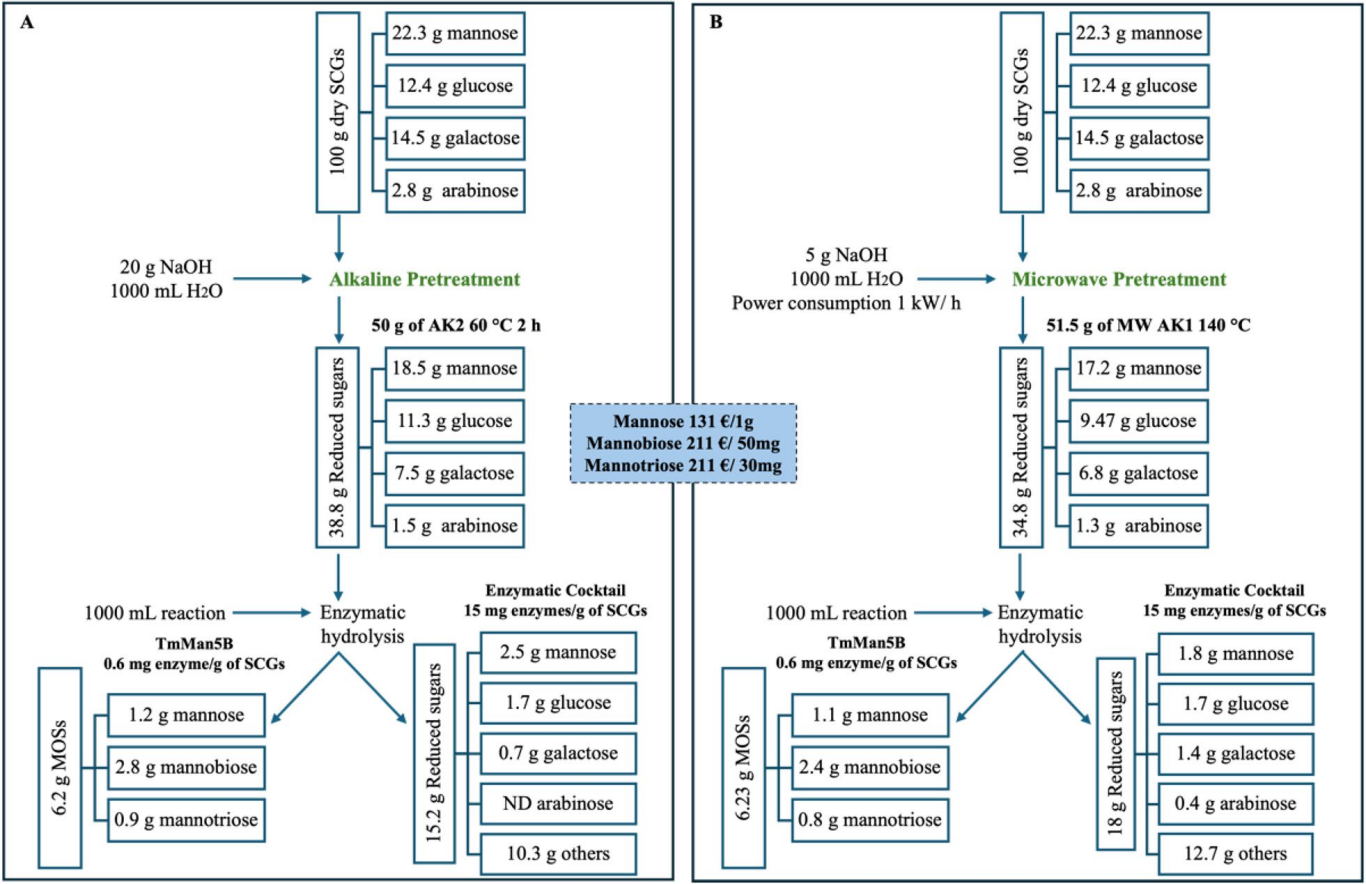
Process mass balance: alkaline pretreatment (**A**) and microwave pretreatment (**B**). All the numbers correspond to g of dry weight

To estimate the process efficiency, a mass balance was evaluated on a 100 g scale by using AK2 at 60 °C 2 h (Fig. 4A) and MW AK1 140 °C (Fig. 4B). After the action of the enzymatic cocktail, 15.2 and 18 g of reducing sugars were obtained, respectively, at the end of the process. The comparison with the total reducing sugars present in the pretreated biomass (38.8 g for AK2 60 °C 2 h and 34.8 for MW AK1 140°, respectively) indicates a conversion yield of more than 50% of the polysaccharides content for both pretreated SCGs. Additionally, the enzymatic conversion of galactomannan by TmMan5B yielded approximately 6 g of MOSs in both processes. The overall mass balance evaluation provides a comprehensive overview of the recovery of high-value sugars from pretreated SCG, highlighting the efficiency of the process. Compared with the mass balance reported in a recent study by Jomnonkhaow et al. [35]. Our study used half the amount of NaOH and a thermostable enzymatic cocktail targeting both cellulose and hemicellulose in SCGs while achieving a nearly identical conversion yield. Our approach is effective in the saccharification process and in producing MOSs while enhancing process sustainability.

## Conclusions

This study highlights the importance of integrating mild pretreatments with specifically designed thermostable polysaccharide-degrading enzymes that target the full range of polysaccharides in SCGs. By developing and applying a targeted enzymatic approach to different pretreated SCG biomasses, we confirmed the versatility and commercial potential of this common waste product as a substrate for producing high-value saccharides, including prebiotic MOS. AK pretreatments, particularly when combined with MW and SC-CO2, were most effective in preserving hemicellulose content while improving enzyme accessibility. In fact, when applied to biomass pretreated under these conditions, the enzymatic cocktail developed in this study demonstrated significantly greater efficiency in hydrolyzing SCG polysaccharides. Moreover, this study highlighted the crucial role of targeted activities aimed at the hydrolysis of hemicelluloses, emphasizing their importance for optimizing saccharide recovery. The potential applications of the derived saccharides are diverse, ranging from functional food ingredients to pharmaceuticals, animal feed, and even biofuels. The proposed approach has proven to be highly effective for SCGs and can contribute to reducing SCG landfill waste and greenhouse gas emissions. It may serve as a proof-of-concept for the valorization of lignocellulosic biomasses. To evaluate the industrial relevance of this process, future research should focus first on an economic analysis to assess production costs, scalability, and market potential of the derived saccharides, particularly considering the growing demand for prebiotic ingredients. Furthermore, cost-optimization strategies, such as using crude enzyme preparations or integrating the process with existing coffee production infrastructure, could help reduce operational expenses. Finally, integrating SCG valorization into biorefinery models, which could incorporate other waste streams, would maximize resource efficiency and strengthen the circular bioeconomy approach.

## Supplementary Information

Additional file 1.

## Data Availability

All data generated or analyzed during this study are included in this published article and its supplementary information files. The materials used and/or analyzed during the current study are available from the corresponding authors on reasonable request.

## Abbreviations

SCGs: Spent coffee grounds
GH: Glycoside hydrolase
CAZy: Carbohydrate active-enzyme database
DW: Distilled water
HT: Hydrothermal pretreatment
AC: Acid pretreatment
AC1: Acid pretreatment with 0.5% H_2_SO_4_
AC2: Acid pretreatment with 2% H_2_SO_4_
AK: Alkaline pretreatment
AK1: Alkaline pretreatment with 0.5% NaOH
AK2: Alkaline pretreatment with 2% NaOH
AK1 30 °C 5 h: AK1 when incubated for 5 h at 30 °C under agitation at 120 rpm
AK2 30 °C 5 h: AK2 when incubated for 5 h at 30 °C under agitation at 120 rpm
AK1 60 °C 2 h: AK1 when incubated for 2 h at 60 °C under agitation at 120 rpm
AK2 60 °C 2 h: AK2 when incubated for 2 h at 60 °C under agitation at 120 rpm
AK1 60 °C 5 h: AK1 when incubated for 5 h at 60 °C under agitation at 120 rpm
AK2 60 °C 5 h: AK2 when incubated for 5 h at 60 °C under 120 rpm
AK1 121 °C 45 min: AK1 when incubated in autoclave of 45 min at 121 °C under 2 bars of pressure
AK2 121 °C 45 min: AK2 with incubation in autoclave of 45 min at 121 °C under 2 bars of pressure
SC-CO_2_: Supercritical CO_2_ extraction
SC-CO_2_E: Supercritical CO_2_ extraction in the presence of 10% ethanol
MW: Microwave pretreatment
MW DW1: MW for 10 min at 140 °C
MW DW2: MW for 10 min at 170 °C
SC-CO_2_ AK1: SC-CO_2_ in the presence of 0.5% NaOH
SC-CO_2_E AK1: SC-CO_2_E in the presence of 0.5% NaOH
SC-CO_2_ AK2: SC-CO_2_ in the presence of 2% NaOH
SC-CO_2_E AK2: SC-CO_2_E in the presence of 2% NaOH
MW AK1 140 °C: MW DW1 in the presence of 0.5% NaOH
MW AK1 170 °C: MW DW2 in the presence of 0.5% NaOH
ASL: Acid-soluble lignin
AIL: Acid-insoluble lignin
MS: Monosaccharides
FT-IR: Fourier transform infrared spectroscopy
CGal: Carob galactomannan
MOSs: Mannooligosaccharides
TmMOS: TmManB5 reaction products from pretreated SCG
RS: Reducing sugars
M2: Mannobiose
M3: Mannotriose

## References

[1] Arevalo-Gallegos A, Ahmad Z, Asgher M, Parra-Saldivar R, Iqbal HMN. Lignocellulose: a sustainable material to produce value-added products with a zero waste approach—a review. Int J Biol Macromol. 2017;99:308–18. 28254573 10.1016/j.ijbiomac.2017.02.097

[2] Lee KH, Jang YW, Lee J, Kim S, Park C, Yoo HY. Statistical optimization of alkali pretreatment to improve sugars recovery from spent coffee grounds and utilization in lactic acid fermentation. Processes. 2021;9(3):494.

[3] Tan ECD, Lamers P. Circular bioeconomy concepts—a perspective. Front Sustain. 2021;2:53.

[4] Abraham A, Mathew AK, Park H, Choi O, Sindhu R, Parameswaran B, et al. Pretreatment strategies for enhanced biogas production from lignocellulosic biomass. Bioresour Technol. 2020;301: 122725. 31958690 10.1016/j.biortech.2019.122725

[5] Yang B, Dai Z, Ding SY, Wyman CE. Enzymatic hydrolysis of cellulosic biomass. Biofuels. 2011;2(4):421–49.

[6] Drula E, Garron ML, Dogan S, Lombard V, Henrissat B, Terrapon N. The carbohydrate-active enzyme database: functions and literature. Nucl Acid Res. 2022;50(D1):D571–7. 10.1093/nar/gkab1045 PMC8728194 34850161

[7] Dumorné K, Córdova DC, Astorga-Eló M, Renganathan P. Extremozymes: a potential source for industrial applications. J Microbiol Biotechnol. 2017;27(4):649–59. 28104900 10.4014/jmb.1611.11006

[8] Iacono R, Strazzulli A, Giglio R, Bitetti F, Cobucci-Ponzano B, Moracci M. Valorization of biomasses from energy crops for the discovery of novel thermophilic glycoside hydrolases through metagenomic analysis. Int J Mol Sci. 2022;23(18):10505. 36142415 10.3390/ijms231810505 PMC9505709

[9] Curci N, Strazzulli A, Iacono R, De Lise F, Maurelli L, Di Fenza M, et al. Xyloglucan oligosaccharides hydrolysis by exo-acting glycoside hydrolases from hyperthermophilic microorganism saccharolobus solfataricus. Int J Mol Sci. 2021;22:7. 10.3390/ijms22073325 PMC8037949 33805072

[10] Murthy PS, Madhava NM. Sustainable management of coffee industry by-products and value addition—a review. Resour Conserv Recycl. 2012;66:45–58.

[11] International Coffee Organization. 2024. 1–12. https://icocoffee.org/. Accessed 3Apr 2024.

[12] The Sustainable Development Goals Report. 2022. https://unstats.un.org/sdgs/report/2022/.

[13] Mensah RQ, Tantayotai P, Rattanaporn K, Chuetor S, Kirdponpattara S, Kchaou M, et al. Properties and applications of green-derived products from spent coffee grounds—steps towards sustainability. Bioresour Technol Rep. 2024;26: 101859.

[14] Obruca S, Benesova P, Kucera D, Petrik S, Marova I. Biotechnological conversion of spent coffee grounds into polyhydroxyalkanoates and carotenoids. New Biotechnol. 2015;32(6):569–74. 10.1016/j.nbt.2015.02.008 25721970

[15] Pereira J, de Melo MMR, Silva CM, Lemos PC, Serafim LS. Impact of a pretreatment step on the acidogenic fermentation of spent coffee grounds. Bioeng. 2022;9(8):362. 10.3390/bioengineering9080362 PMC9404928 36004887

[16] Moreira ASP, Nunes FM, Domingues MRM, Coimbra MA. Galactomannans in coffee. In: Moreira ASP, Nunes FM, Domingues MRM, Coimbra MA, editors. Coffee in health and disease prevention. Amsterdam: Elsevier Inc.; 2014. p. 173–83.

[17] Portillo OR, Arévalo AC. Coffee’s carbohydrates. A critical review of scientific literature. Bionatura. 2022. 10.21931/RB/2022.07.03.11.

[18] De Oliveira Petkowicz CL. Polysaccharides in coffee and their relationship to health: an overview. In: De Oliveira Petkowicz CL, editor. Coffee in health and disease prevention. Amsterdam: Elsevier Inc.; 2014. p. 163–72.

[19] Knoch E, Dilokpimol A, Geshi N. Arabinogalactan proteins: focus on carbohydrate active enzymes. Front Plant Sci. 2014;5:198. 24966860 10.3389/fpls.2014.00198 PMC4052742

[20] de Melo MMR, Barbosa HMA, Passos CP, Silva CM. Supercritical fluid extraction of spent coffee grounds: measurement of extraction curves, oil characterization and economic analysis. J Supercrit Fluid. 2014;86:150–9.

[21] Binod P, Satyanagalakshmi K, Sindhu R, Janu KU, Sukumaran RK, Pandey A. Short duration microwave assisted pretreatment enhances the enzymatic saccharification and fermentable sugar yield from sugarcane bagasse. Renew Energ. 2012;37(1):109–16.

[22] Sluiter A, Hames B, Ruiz R, Scarlata C, Sluiter J, Templeton D, et al. Determination of structural carbohydrates and lignin in biomass: laboratory analytical procedure (LAP). 2008. http://www.nrel.gov/biomass/analytical_procedures.html. Accessed 17 Mar 2022.

[23] Rizwan AB, Hasmukh AM. Extraction of oligosaccharides and phenolic compounds by roasting pretreatment and enzymatic hydrolysis from spent coffee ground. J Appl Biol Biotechnol. 2020;8(4):75–81.

[24] Pouwels J, Moracci M, Cobucci-Ponzano B, Perugino G, Van Der Oost J, Kaper T, et al. Activity and stability of hyperthermophilic enzymes: a comparative study on two archaeal-glycosidases. Extremophiles. 2000;4:157–64. 10879560 10.1007/s007920070030

[25] Cobucci-Ponzano B, Zorzetti C, Strazzulli A, Carillo S, Bedini E, Corsaro MM, et al. A novel α-d-galactosynthase from thermotoga maritima converts β-d-galactopyranosyl azide to α-galacto-oligosaccharides. Glycobiology. 2011;21(4):448–56. 21084405 10.1093/glycob/cwq177

[26] Comfort DA, Bobrov KS, Ivanen DR, Shabalin KA, Harris JM, Kulminskaya AA, et al. Biochemical analysis of thermotoga maritima GH36 α-galactosidase (TmGalA) confirms the mechanistic commonality of clan GH-D glycoside hydrolases. Biochemistry. 2007;46(11):3319–30. 17323919 10.1021/bi061521n

[27] Morana A, Paris O, Maurelli L, Rossi M, Cannio R. Gene cloning and expression in *Escherichia coli* of a bi-functional β-D-xylosidase/α-L-arabinosidase from sulfolobus solfataricus involved in xylan degradation. Extremophiles. 2007;11(1):123–32. 17033733 10.1007/s00792-006-0020-7

[28] Zhang M, Jiang Z, Li L, Katrolia P. Biochemical characterization of a recombinant thermostable β-mannosidase from thermotoga maritima with transglycosidase activity. J Mol Catal B Enzym. 2009;60(3):119–24.

[29] Branda SS, González-Pastor JE, Dervyn E, Ehrlich SD, Losick R, Kolter R. Genes involved in formation of structured multicellular communities by bacillus subtilis. J Bacteriol. 2004;186(12):3970–9. 15175311 10.1128/JB.186.12.3970-3979.2004 PMC419949

[30] Vittoria M, Saggese A, Isticato R, Baccigalupi L, Ricca E. Probiotics as an alternative to antibiotics: genomic and physiological characterization of aerobic spore formers from the human intestine. Microorganisms. 2023;11(8). https://www.ncbi.nlm.nih.gov/pmc/articles/PMC10458579/. Accessed 11 May 2024 10.3390/microorganisms11081978 PMC10458579 37630538

[31] Fakhry S, Manzo N, D’Apuzzo E, Pietrini L, Sorrentini I, Ricca E, et al. Characterization of intestinal bacteria tightly bound to the human ileal epithelium. Res Microbiol. 2009;160(10):817–23. 19782749 10.1016/j.resmic.2009.09.009

[32] Magengelele M, Malgas S, Pletschke B. IB. Bioconversion of spent coffee grounds to prebiotic mannooligosaccharides—an example of biocatalysis in biorefinery. RSC Adv. 2023;13(6):3773–80. 36756573 10.1039/d2ra07605e PMC9890647

[33] Castaldi S, Valkov VT, Ricca E, Chiurazzi M, Isticato R. Use of halotolerant *Bacillus amyloliquefaciens* RHF6 as a bio-based strategy for alleviating salinity stress in Lotus japonicus cv Gifu. Microbiol Res. 2023;268: 127274. 36527786 10.1016/j.micres.2022.127274

[34] Mussatto SI, Carneiro LM, Silva JPA, Roberto IC, Teixeira JA. A study on chemical constituents and sugars extraction from spent coffee grounds. Carbohydr Polym. 2011;83(2):368–74.

[35] Jomnonkhaow U, Plangklang P, Reungsang A, Peng CY, Chu CY. Valorization of spent coffee grounds through integrated bioprocess of fermentable sugars, volatile fatty acids, yeast-based single-cell protein and biofuels production. Bioresour Technol. 2024;393: 130107. 38016585 10.1016/j.biortech.2023.130107

[36] López-Linares JC, García-Cubero MT, Coca M, Lucas S. Efficient biobutanol production by acetone–butanol–ethanol fermentation from spent coffee grounds with microwave assisted dilute sulfuric acid pretreatment. Bioresour Technol. 2021;320: 124348. 33190095 10.1016/j.biortech.2020.124348

[37] Nguyen QA, Cho EJ, Lee DS, Bae HJ. Development of an advanced integrative process to create valuable biosugars including manno-oligosaccharides and mannose from spent coffee grounds. Bioresour Technol. 2019;272:209–16. 30340187 10.1016/j.biortech.2018.10.018

[38] Ravindran R, Jaiswal S, Abu-Ghannam N, Jaiswal AK. Evaluation of ultrasound assisted potassium permanganate pre-treatment of spent coffee waste. Bioresour Technol. 2017;224:680–7. 27866804 10.1016/j.biortech.2016.11.034

[39] Zheng Y, Lee C, Yu C, Cheng YS, Zhang R, Jenkins BM, et al. Dilute acid pretreatment and fermentation of sugar beet pulp to ethanol. Appl Energy. 2013;105:1–7.

[40] Kim JS, Lee YY, Kim TH. A review on alkaline pretreatment technology for bioconversion of lignocellulosic biomass. Bioresour Technol. 2016;199:42–8. 26341010 10.1016/j.biortech.2015.08.085

[41] da Silva RPFF, Rocha-Santos TAP, Duarte AC. Supercritical fluid extraction of bioactive compounds. TrAC Trend Anal Chem. 2016;76:40–51.

[42] Ravindran R, Jaiswal S, Abu-Ghannam N, Jaiswal AK. A comparative analysis of pretreatment strategies on the properties and hydrolysis of brewers’ spent grain. Bioresour Technol. 2018;248:272–9. 28648256 10.1016/j.biortech.2017.06.039

[43] Ballesteros LF, Teixeira JA, Mussatto SI. Chemical, functional, and structural properties of spent coffee grounds and coffee silverskin. Food Bioprocess Technol. 2014;7(12):3493–503.

[44] Ravindran R, Jaiswal S, Abu-Ghannam N, Jaiswal AK. Two-step sequential pretreatment for the enhanced enzymatic hydrolysis of coffee spent waste. Bioresour Technol. 2017;239:276–84. 28531852 10.1016/j.biortech.2017.05.049

[45] Tsafrakidou P, Moutsoglou A, Prodromidis P, Moschakis T, Goula A, Biliaderis CG, et al. Aqueous ammonia soaking pretreatment of spent coffee grounds for enhanced enzymatic hydrolysis: a bacterial cellulose production application. Sustain Chem Pharm. 2023;33: 101121.

[46] Tripathi S, Murthy PS. Coffee oligosaccharides and their role in health and wellness. Food Res Int. 2023;173: 113288. 37803601 10.1016/j.foodres.2023.113288

[47] Campos-Vega R, Loarca-Piña G, Vergara-Castañeda HA, Dave OB. Spent coffee grounds: a review on current research and future prospects. Trend Food Sci Technol. 2015;45(1):24–36.

[48] Jönsson LJ, Alriksson B, Nilvebrant NO. Bioconversion of lignocellulose: inhibitors and detoxification. Biotechnol Biofuels. 2013;6(1):16. 23356676 10.1186/1754-6834-6-16 PMC3574029

[49] Pan X, Wu T, Zhang L, Cai L, Song Z. Influence of oligosaccharides on the growth and tolerance capacity of lactobacilli to simulated stress environment. Lett Appl Microbiol. 2009;48(3):362–7. 19187509 10.1111/j.1472-765X.2008.02539.x

[50] Landini P, Antoniani D, Burgess JG, Nijland R. Molecular mechanisms of compounds affecting bacterial biofilm formation and dispersal. Appl Microbiol Biotechnol. 2010;86(3):813–23. 20165945 10.1007/s00253-010-2468-8

[51] Yin J, Peng X, Yang A, Lin M, Ji K, Dai X, et al. Impact of oligosaccharides on probiotic properties and B vitamins production: a comprehensive assessment of probiotic strains. Int J Food Sci Technol. 2024;59(9):6044–64.

[52] Kumar Suryawanshi R, Kango N. Production of mannooligosaccharides from various mannans and evaluation of their prebiotic potential. Food Chem. 2021;334: 127428. 32688173 10.1016/j.foodchem.2020.127428

[53] Magengelele M, Hlalukana N, Malgas S, Rose SH, van Zyl WH, Pletschke BI. Production and in vitro evaluation of prebiotic manno-oligosaccharides prepared with a recombinant *Aspergillus niger* endo-mannanase, Man26A. Enzym Microb Technol. 2021;150: 109893. 10.1016/j.enzmictec.2021.109893 34489046

