# Supplementary material for: Carbohydrate conversion in spent coffee grounds: pretreatment strategies and novel enzymatic cocktail to produce value-added saccharides and prebiotic mannooligosaccharides
Source: Biotechnol Biofuels Bioprod. 2025 Jan 7;18:2. doi: 10.1186/s13068-024-02601-6 (PMC11705863; doi:10.1186/s13068-024-02601-6)
Supplement: Supplementary file 1 — Additional file 1. [file 13068_2024_2601_MOESM1_ESM.docx]

**Carbohydrate Conversion in Spent Coffee Grounds: Pretreatment Strategies and Novel Enzymatic Cocktail to Produce Value-Added Saccharides and Prebiotic Mannooligosaccharides**

**Table S1.** List of the enzymes tested for the degradation of the targeted polysaccharides in SCGs

| **Target Polysaccharide** | **Enzyme** | **Code** | **GH family** | **Source** |
| --- | --- | --- | --- | --- |
| **Galactomannan** | Endo β-1,4-mannanase | TmMan5B | 5 | Nzytech |
|  | Endo β-1,4-mannanase | CtMan5A | 5 | Nzytech |
|  | β-mannosidase | TmManA | 2 | In-house^*^ |
|  | α-1,6-galactosidase | TmGalA | 36 | In-house^*^ |
| **Arabinogalactan II** | Endo β-1,3 galactanase | Gal3A | 16 | Novonesis |
|  | Endo β-1,3 galactanase | Gal3B | 16 | Novonesis |
|  | Endo β-1,3 galactanase | Gal3C | 16 | Novonesis |
|  | Endo β-1,3 galactanase | Gal3D | 16 | Novonesis |
|  | Endo β-1,3 galactanase | Gal3E | 16 | Novonesis |
|  | Endo β-1,3 galactanase | Gal3F | 16 | Novonesis |
|  | Endo β-1,6 galactanase | Gal6A | 30 | Novonesis |
|  | Endo β-1,6 galactanase | Gal6B | 30 | Novonesis |
|  | Endo β-1,6 galactanase | Gal6C | 30 | Novonesis |
|  | Endo β-1,6 galactanase | Gal6D | 5 | Novonesis |
|  | Exo β-1,3 galactanase | Exo Gal6 | 43 | Novonesis |
|  | β-1,3-galactosidase | CelB | 1 | In-house^*^ |
|  | α-arabinosidase | XarS | 3 | In-house^*^ |
| **Cellulose** | Cellulase | 12A | 12 | Nzytech |
|  | Cellobiohydrolase | CBHI | 7 | Megazyme |
|  | β-1,4-glucosidase | CelB | 1 | In-house^*^ |

*: Enzymes expressed and purified in our laboratory, as stated in the literature related to each enzyme.

**Table S2.** The selected enzymes, their specific activity, and the concentration used in the enzymatic cocktail.

| **Enzyme** | **GH** | **Substrate** | **Concentration** | **Specific Activity (U/mg)** | **µg used in cocktail/mL** | **U used in cocktail/ mL** |
| --- | --- | --- | --- | --- | --- | --- |
| **TmMan5B** | 5 | Carob Galactomannan | 1 % (w/v) | 30 | 30 | 0.9 |
| **TmGalA** | 36 | 4mM 4-Nitrophenyl α-D-galactopyranoside | 4 mM | 37 | 20 | 0.74 |
| **TmManA** | 2 | 4-Nitrophenyl-β-D-mannopyranoside | 5 mM | 9 | 30 | 0.27 |
| **CelB** | 1 | 4-Nitrophenyl β-d-galactopyranoside | 5 mM | 45 | 40 | 1.8 |
| **XarS** | 3 | 4-Nitrophenyl-α-L-arabinofuranoside | 1.5 mM | 8 | 10 | 0.08 |
| **12A** | 12 | Carboxymethyl Cellulose (CMC) | 0.5 % (w/v) | 4 | 40 | 0.16 |
| **Gal3D** | 16 | Acid hydrolyzed Larch Arabinogalactan | 1 % (w/v) | 1 | 40 | 0.04 |
| **Gal6D** | 5 | Larch Arabinogalactan II | 1 % (w/v) | 2.5 | 40 | 0.1 |
| **CBHI** | 7 | ND^a^ | ND^a^ | ND^a^ | 500 | 0.05 |
| **Total** |  |  |  |  | 750 |  |

The enzymes were individually tested for activity on the appropriate substrates at 50 °C in 100 mM sodium acetate buffer, pH 5.5. Activity values are indicated as U/mg. ^a^ND, not determined; the specific activity of CBHI is 0.1 U/mg on 4NP-β-lactoside at 40°C, in sodium acetate buffer pH 4.5 (Megazyme).

**Table S3.** The percentage of solid yield, sugar recovery, and delignification of pretreated SCGs after each pretreatment.

| **Sample** | **Solid yield^a^ %** | **Sugar recovery^b^ %** | **Delignification^c^ %** |
| --- | --- | --- | --- |
| **Raw SCGs** | - | - | - |
| **HT** | 79.5 | 62.5 | 21.5 |
| **AC1** | 57.0 | 39.4 | 16.2 |
| **AC2** | 47.5 | 25.1 | 20.7 |
| **AK1 30 °C 5 h** | 60.5 | 50.5 | 53.9 |
| **AK2 30 °C 5 h** | 52.5 | 71.7 | 62.9 |
| **AK1 60 °C 2 h** | 59.0 | 49.0 | 59.6 |
| **AK2 60 °C 2 h** | 50.0 | 74.5 | 71.4 |
| **AK1 60 °C 5 h** | 48.0 | 59.6 | 71.4 |
| **AK2 60 °C 5 h** | 41.5 | 56.0 | 81.6 |
| **AK1 121 °C 45** | 38.5 | 42.7 | 75.6 |
| **AK2 121 °C 45** | 22.5 | 28.2 | 92.9 |
| **SC-CO_2_** | 92.0 | 81.4 | 19.5 |
| **SC-CO_2_E** | 84.0 | 53.3 | 32.0 |
| **SC-CO_2_ AK1** | 57.5 | 76.3 | 61.6 |
| **SC-CO_2_E AK1** | 59.0 | 77.8 | 63.9 |
| **SC-CO_2_ AK2** | 52.5 | 67.8 | 72.4 |
| **SC-CO_2_E AK2** | 44.5 | 57.6 | 78.7 |
| **MW DW 140 °C** | 80.5 | 90.4 | 26.5 |
| **MW DW 170 °C** | 71.0 | 69.9 | 37.2 |
| **MW AK1 140 °C** | 51.5 | 67.1 | 64.5 |
| **MW AK1 170 °C** | 51.5 | 60.1 | 69.5 |

^a^ $\frac{The dry weight of SCGs after pretreatment}{The dry weight of starting SCGs}\times100$

^b^ $\frac{The percentage of total sugar in pretreated SCGs (Table 1)}{The percentage of total sugar in raw SCGs (Table 1)}\times Solid yield \%$

^c^  100 $-\frac{The percentage of total lignin in pretreated SCGs (Table 1)}{The percentage of total lignin in raw SCGs (Table 1)}\times Solid yield \%$

**Table S4.** TmMan5B reaction products analyses.

|  | **Sugars released (mg/mL)** | | |  |
| --- | --- | --- | --- | --- |
|  | **SC-CO_2_E AK2**  **Small scale** | **AK2 60 °C 2 h**  **Small scale** | **MW AK1 140 °C**  **Small scale** | **MW AK1 140 °C Scaled up** |
| **Mannose** | 0.9 ± 0.1 | 1.1 ± 0.1 | 1.0 ± 0.1 | 1.2 ± 0.1 |
| **Mannobiose** | 1.6 ± 0.1 | 2.4 ± 0.1 | 2.4 ± 0.2 | 2.8 ± 0.2 |
| **Mannotriose** | 1.4 ± 0.1 | 0.8 ± 0.0 | 0.8 ± 0.0 | 0.9 ± 0.1 |
| **Mannotetraose** | ND | ND | ND | ND |
| **Mannopentaose** | ND | ND | ND | ND |
| **Mannohexaose** | ND | ND | ND | ND |
| **Total RS** | 5.26 ± 0.4 | 5.9 ± 0.48 | 5.56 ± 0.25 | 6.23 ± 0.36 |

MW: microwave, AK1: 0.5 % NaOH, SC-CO_2_E: supercritical CO_2_ + 10 % ethanol, AK2: 2.0 % NaOH, ND: Not Detected. Mannose, Mannobiose and Mannotriose were identified and quantified by HPAEC-PAD. Total RS: total reducing sugars determined by Somogyi-Nelson method.

**Table S5.** Generation time (h) of the five bacterial strains used in the study.

| **Bacterial strains** | **Generation Time (h)** | | | | | |
| --- | --- | --- | --- | --- | --- | --- |
|  | **CTRL** | **RB** | **+ 0.2 %**  **Glucose** | **+ 0.2 %  TmMOS** | **+ 0.5 % Glucose** | **+ 0.5 %  TmMOS** |
| ***L. rhamnosus* ATCC53103** | 0 | 0 | 2 | 2.5 | 2 | 2 |
| ***L. gasseri* SF1183** | 0 | 0 | 4 | 3 | 3 | 3.5 |
| ***B. subtilis* NCIB3610** | 0 | 0 | 1 | 1 | 1.5 | 2.5 |
| ***B. velenzensis* MV4** | 0 | 0 | 1.5 | 1 | 1 | 1.5 |
| ***P. megaterium* MV30** | 0 | 0 | 6 | 1.5 | 2 | 2 |

CRTL: control, RB: enzymatic hydrolysis reaction blank (no enzymes), TmMOS: reaction product

of SCGs MW AK1 140 °C hydrolysis by TmManB5.


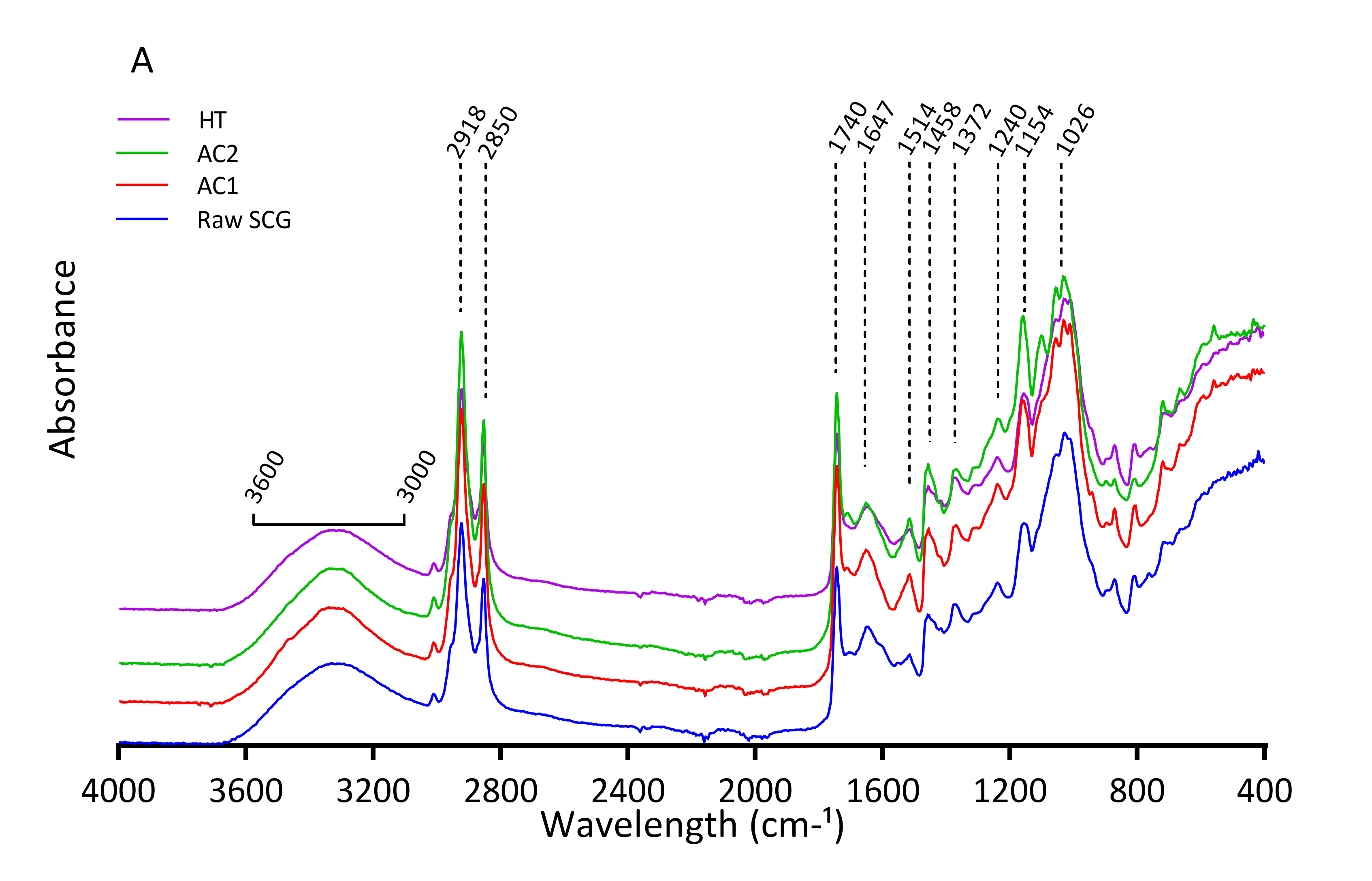

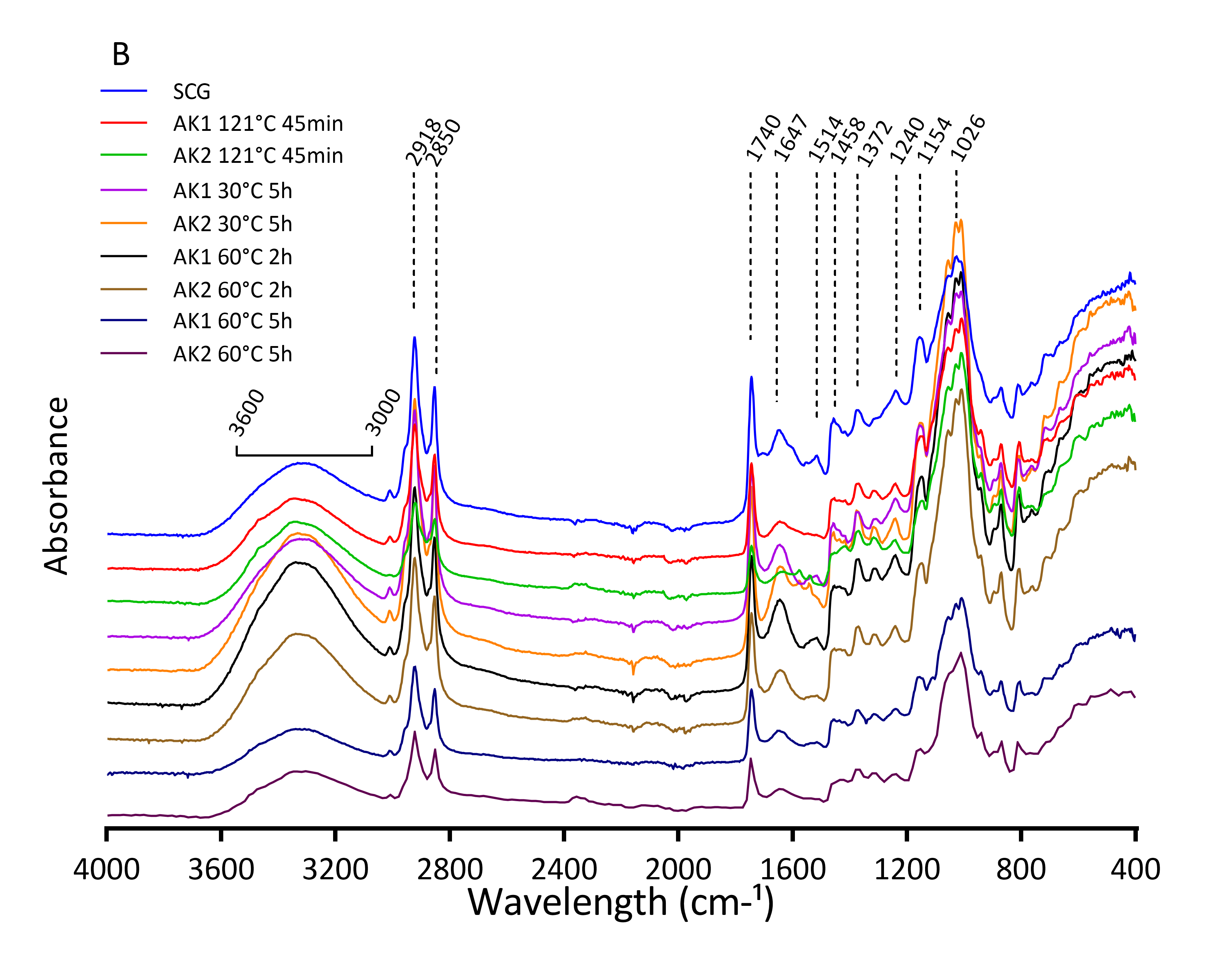

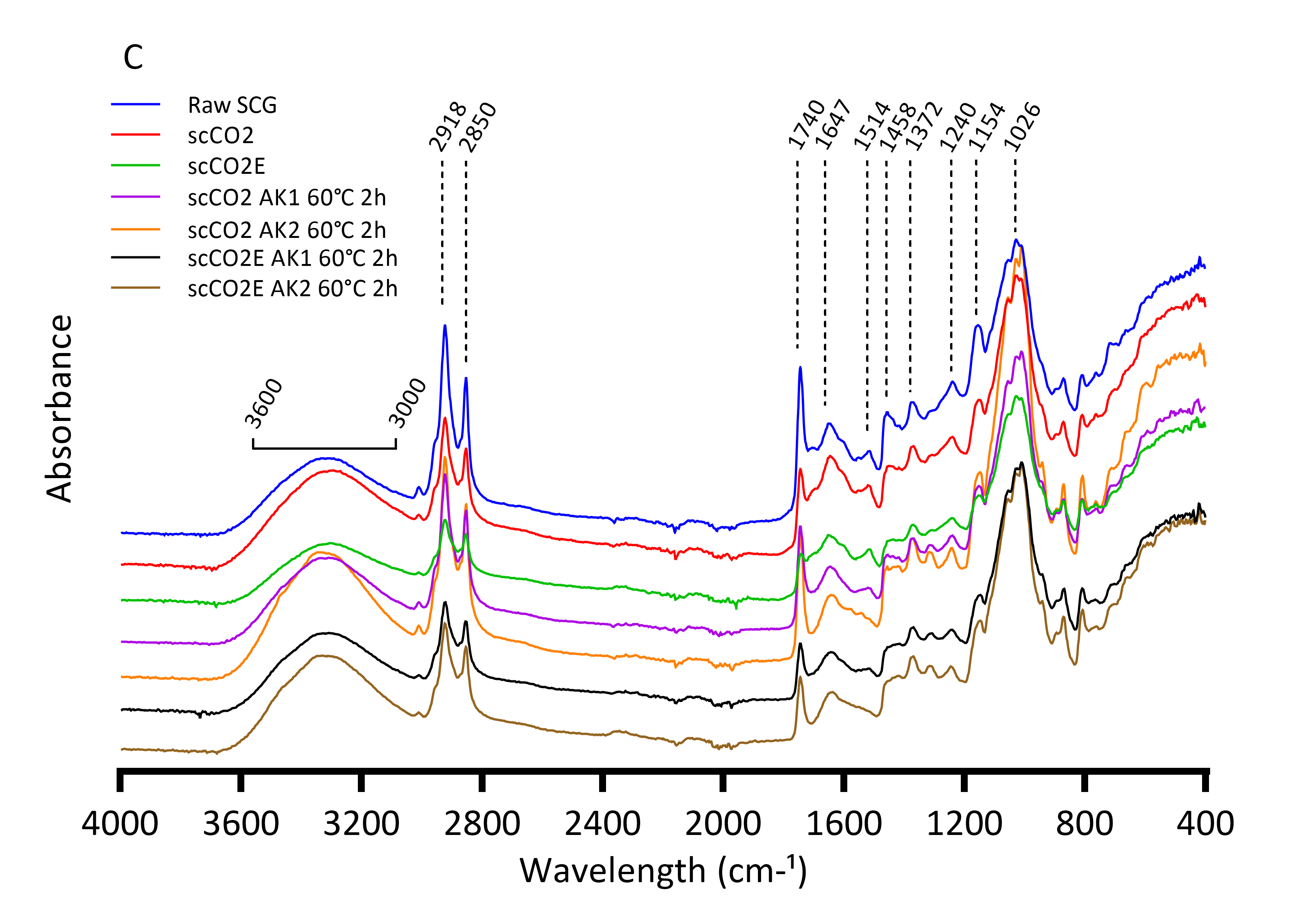

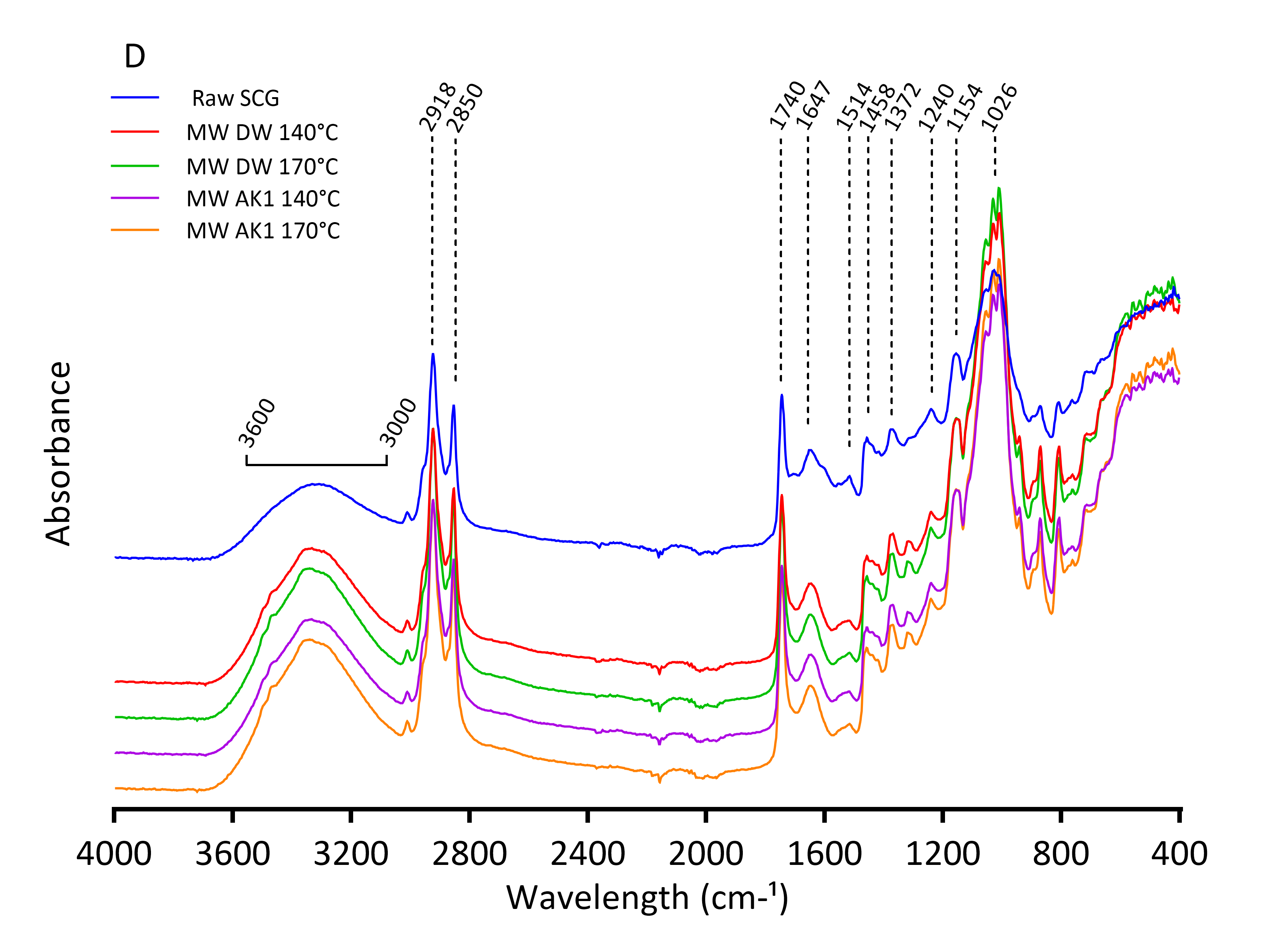


**Figure S1.** FT-IR absorbance spectra profile at 400-4000 cm⁻¹ wavenumber range for SCGs derived from (A) HT (Hydrothermal pretreatment), 0.5 % and 2.0 % H_2_SO_4_ (AC1 and AC2 pretreatment), (B) 0.5 % and 2.0 % NaOH (AK1, and AK2 pretreatments), (C) supercritical extraction (SC-CO_2_), combined with 0.5 % and 2.0 % NaOH (AK1, and AK2 pretreatment), and (D) Microwave pretreatment (MW), with DW (distilled water) or AK1 (0.5 % NaOH) compared with raw SCGs.

**
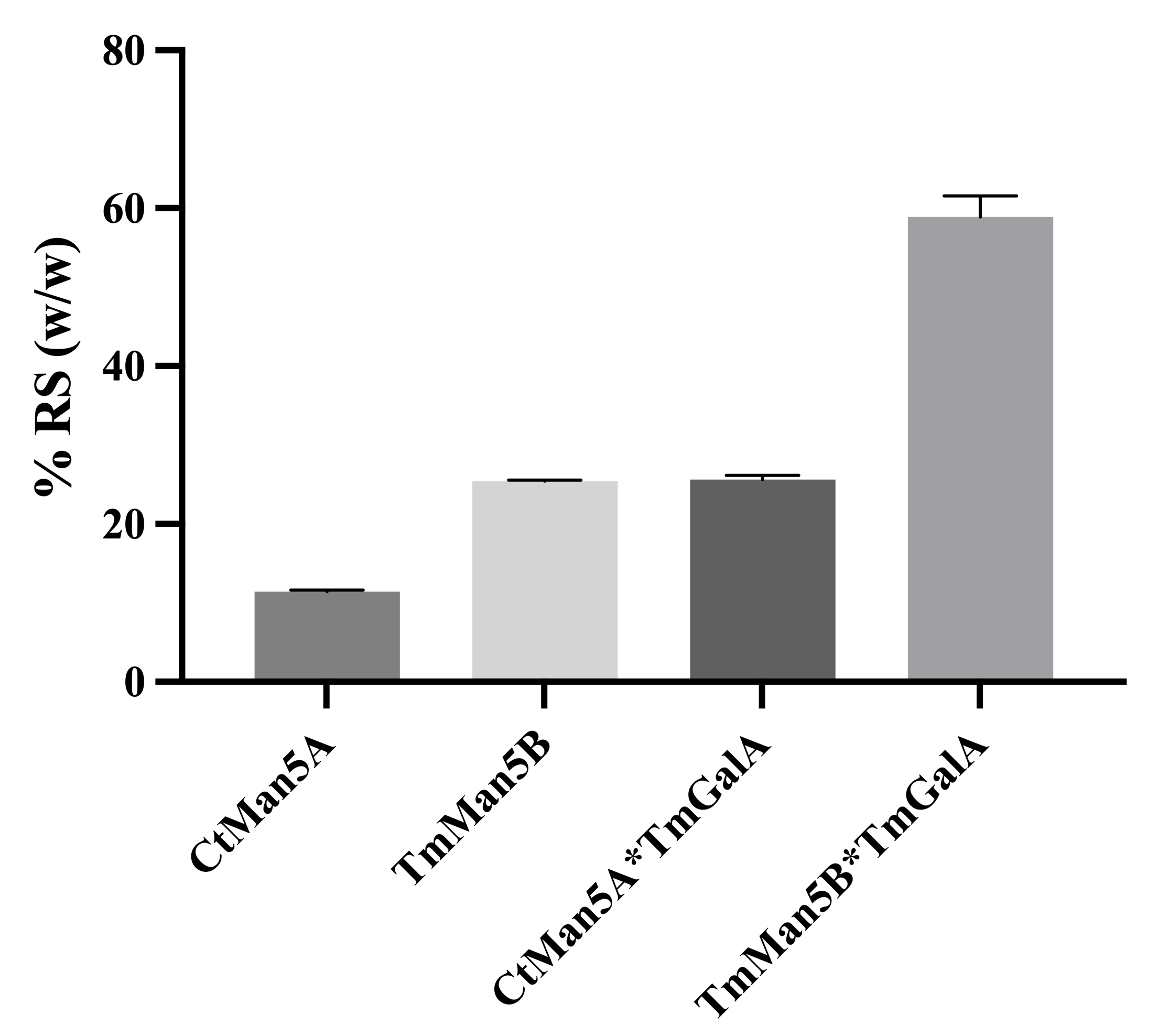
**

**Figure S2.** The hydrolysis of carob galactomannan was compared using endo β-1,4-mannanase from *Clostridium thermocellum* (CtMan5A, Nzytech) and *Thermotoga maritima* (TmMan5B, Nzytech), both individually and in combination with α-1,6-galactosidase from *Thermotoga maritima* (TmGalA). The efficiency of hydrolysis was evaluated by measuring the reducing sugars (RS) and expressed as a percentage of dry weight (% RS, w/w) after 16 hours at 50 °C.

**Figure S3.** The hydrolysis of Larch arabinogalactan II was compared using endo β-1,3-galactanases (Gal3A-E) and endo β-1,6-galactanases (Gal6A-D) both individually (A) and in combination (B). Further on, the best combination was tested with the enzymes Exo-Gal6, CelB, and XarS (C). The efficiency of hydrolysis was evaluated by measuring the reducing sugars (RS) and expressed as a percentage of dry weight (% RS, w/w) after 16 hours at 50 °C.


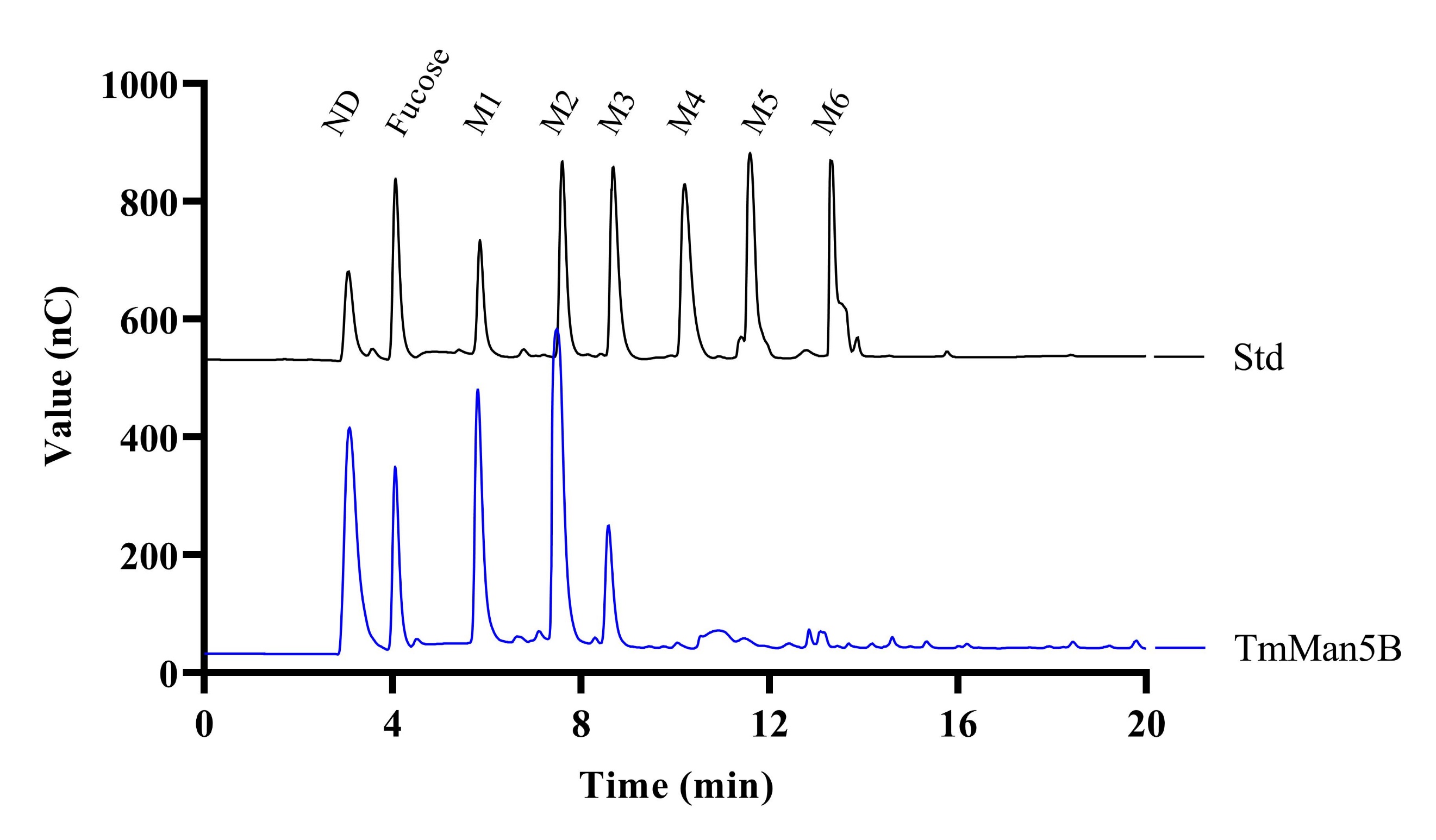


**Figure S4.** The HPAEC-PAD profile of the MOSs was identified and quantified after the enzymatic hydrolysis of MW AK1 140 °C pretreated SCGs. TmMan5B: (GH5) endo β-mannanase, std: standard, M1 to M6: mannobiose to mannohexaose, ND: not defined.


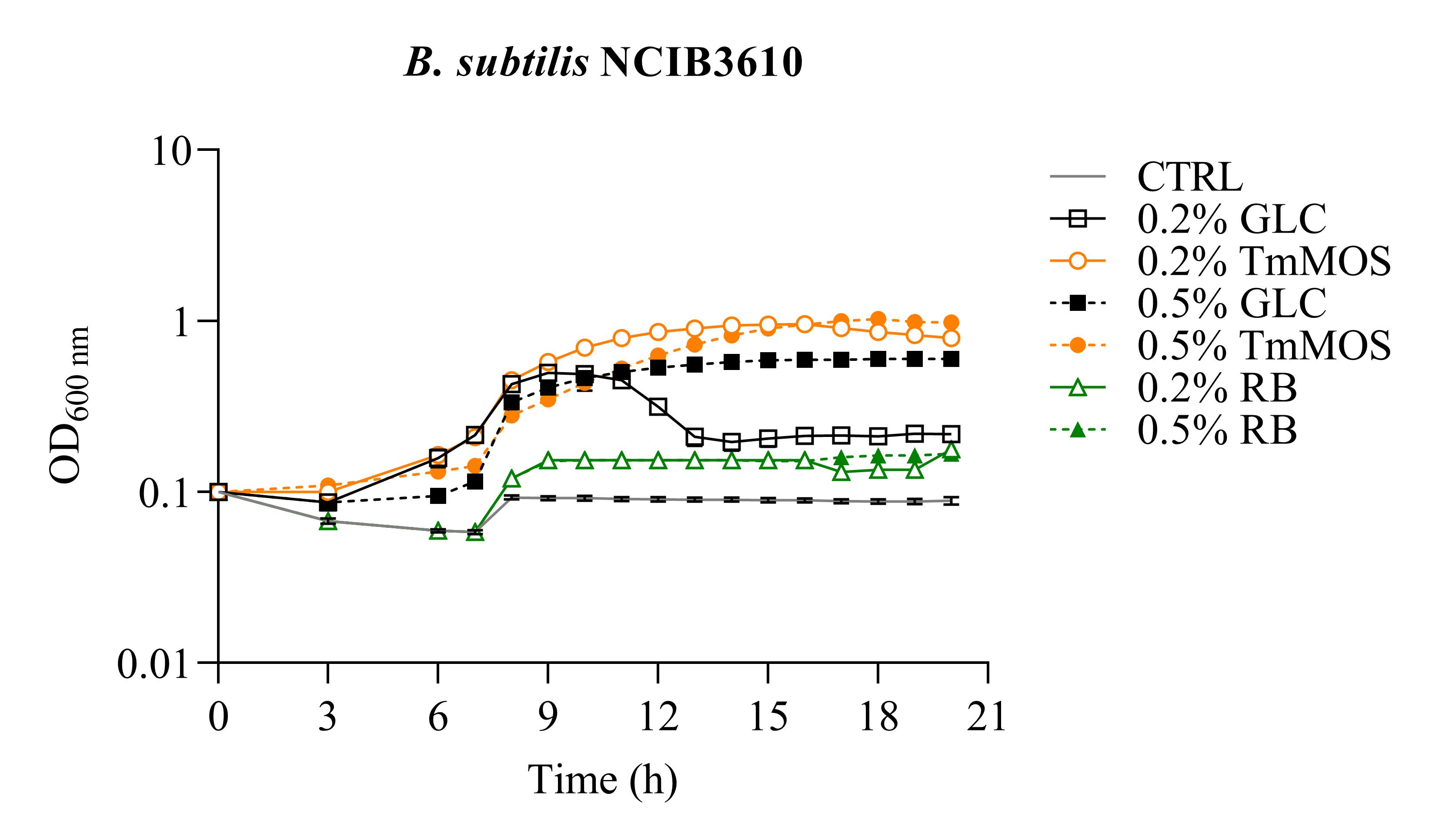

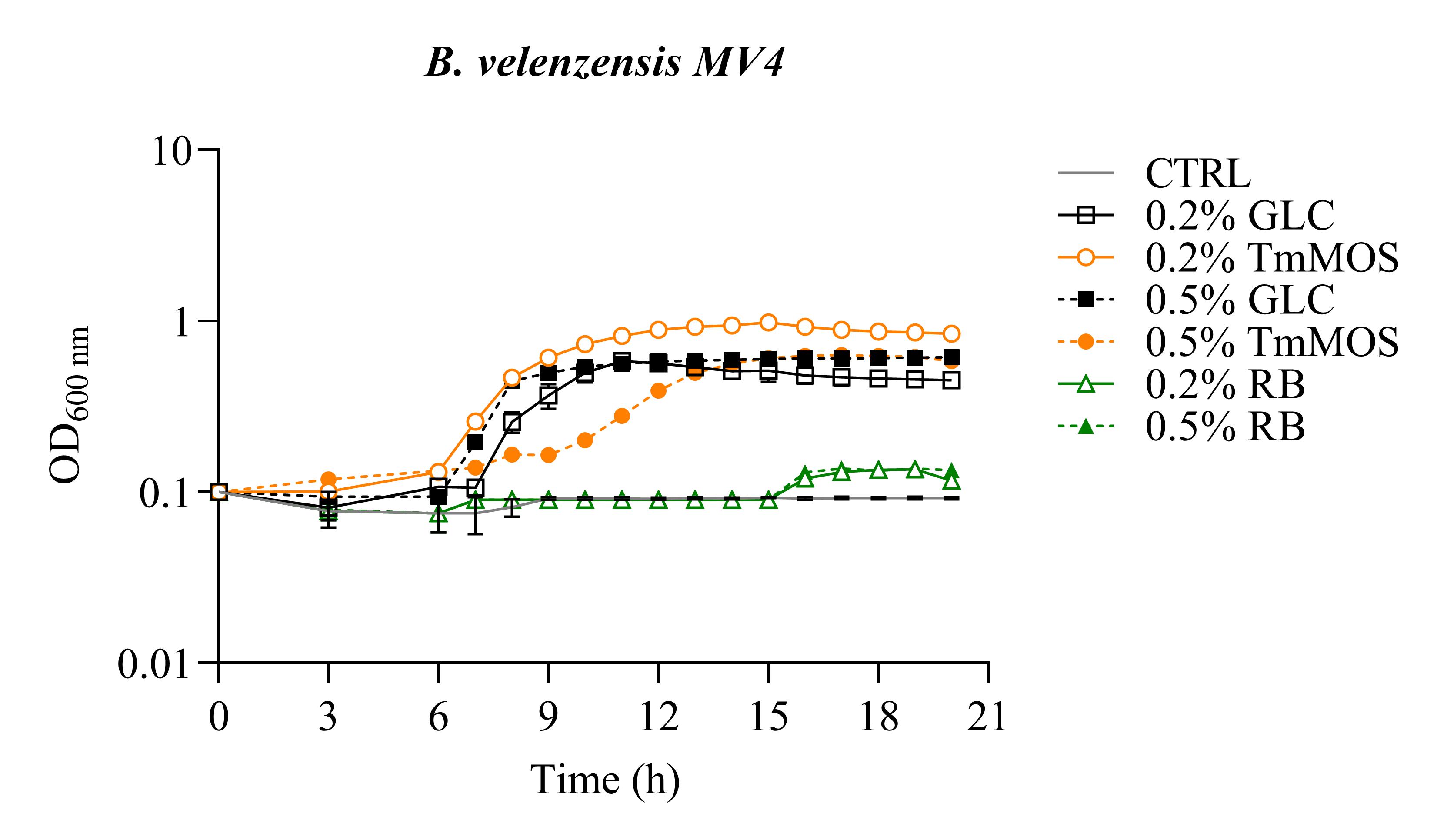

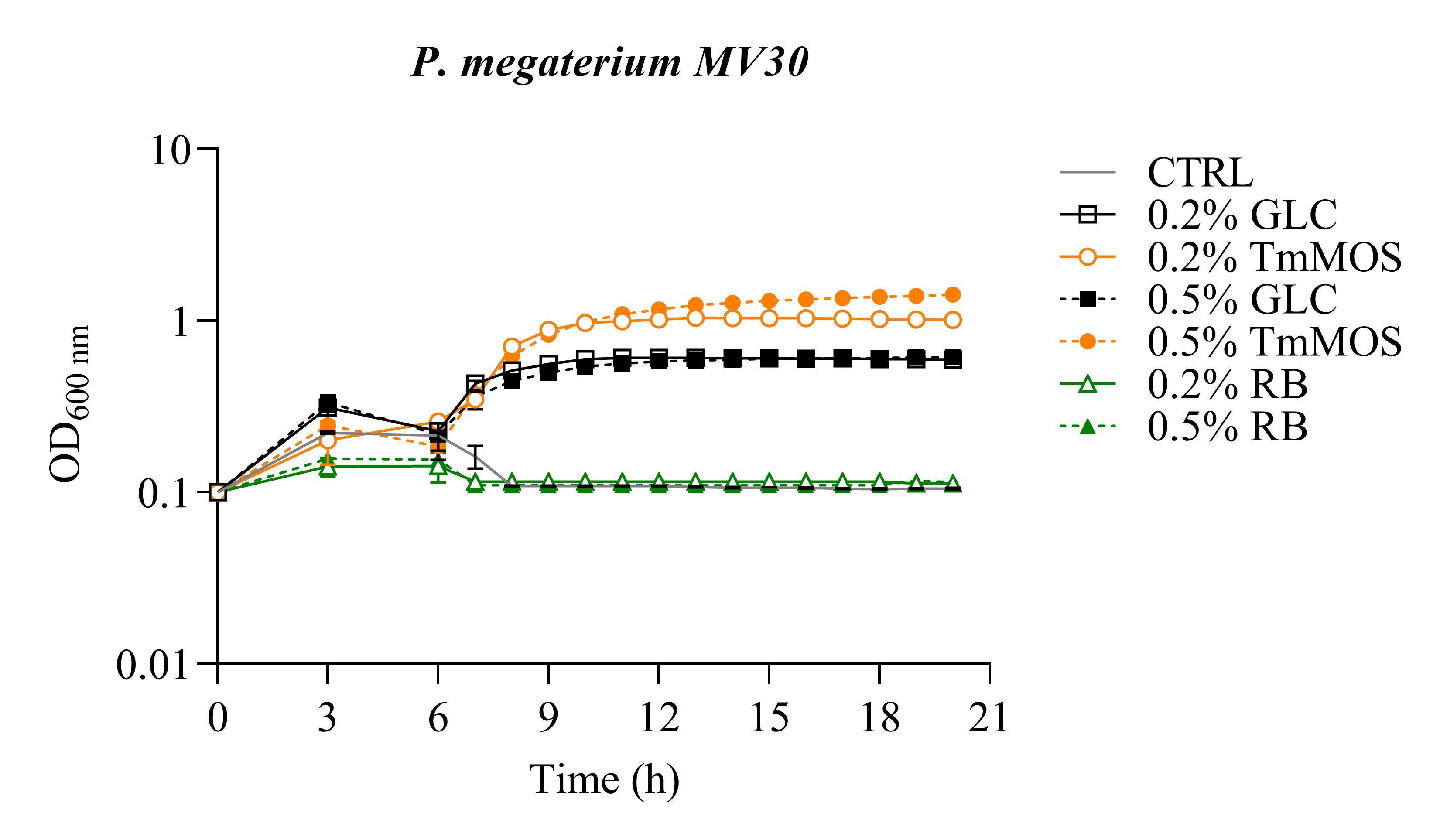

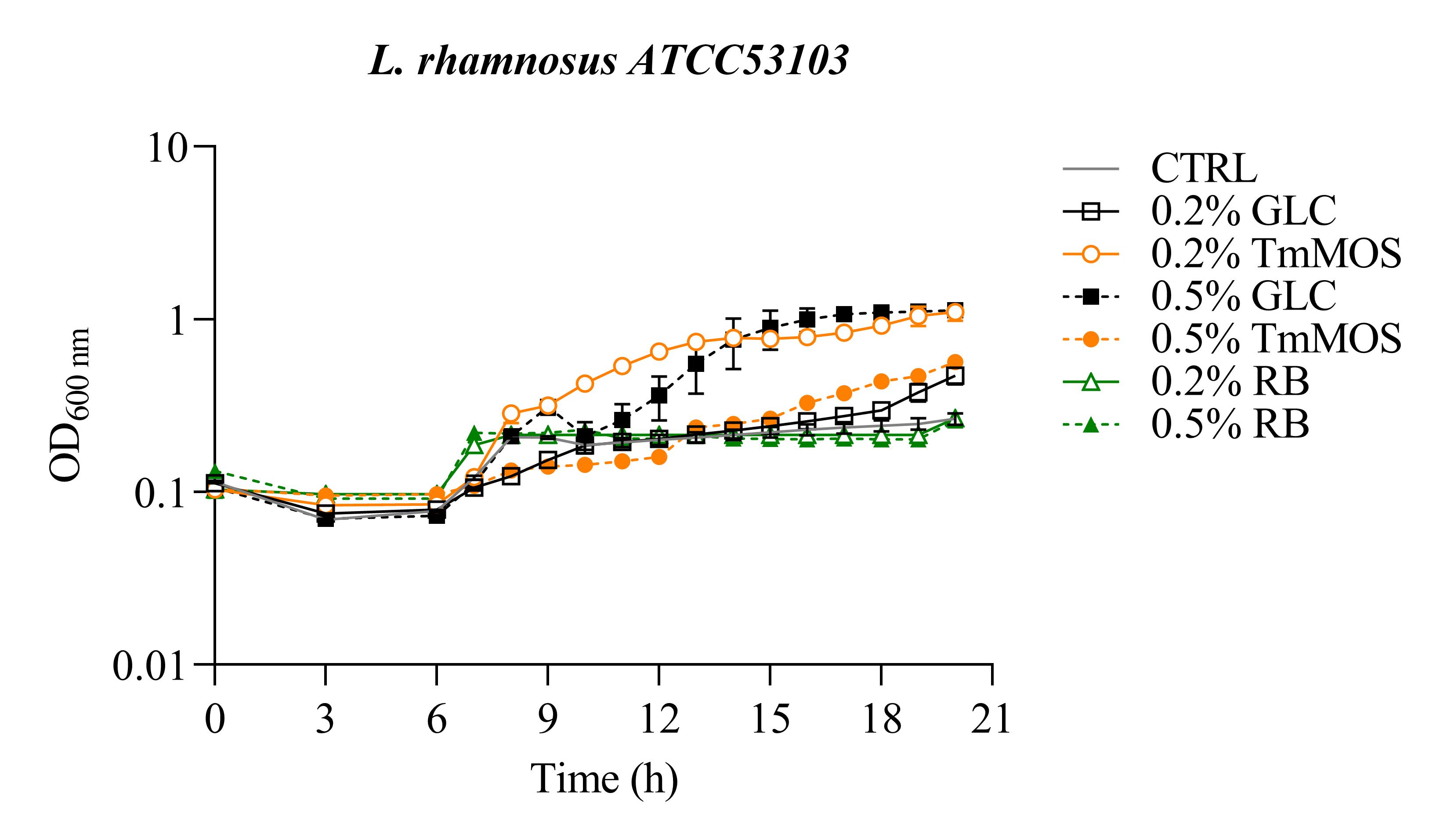

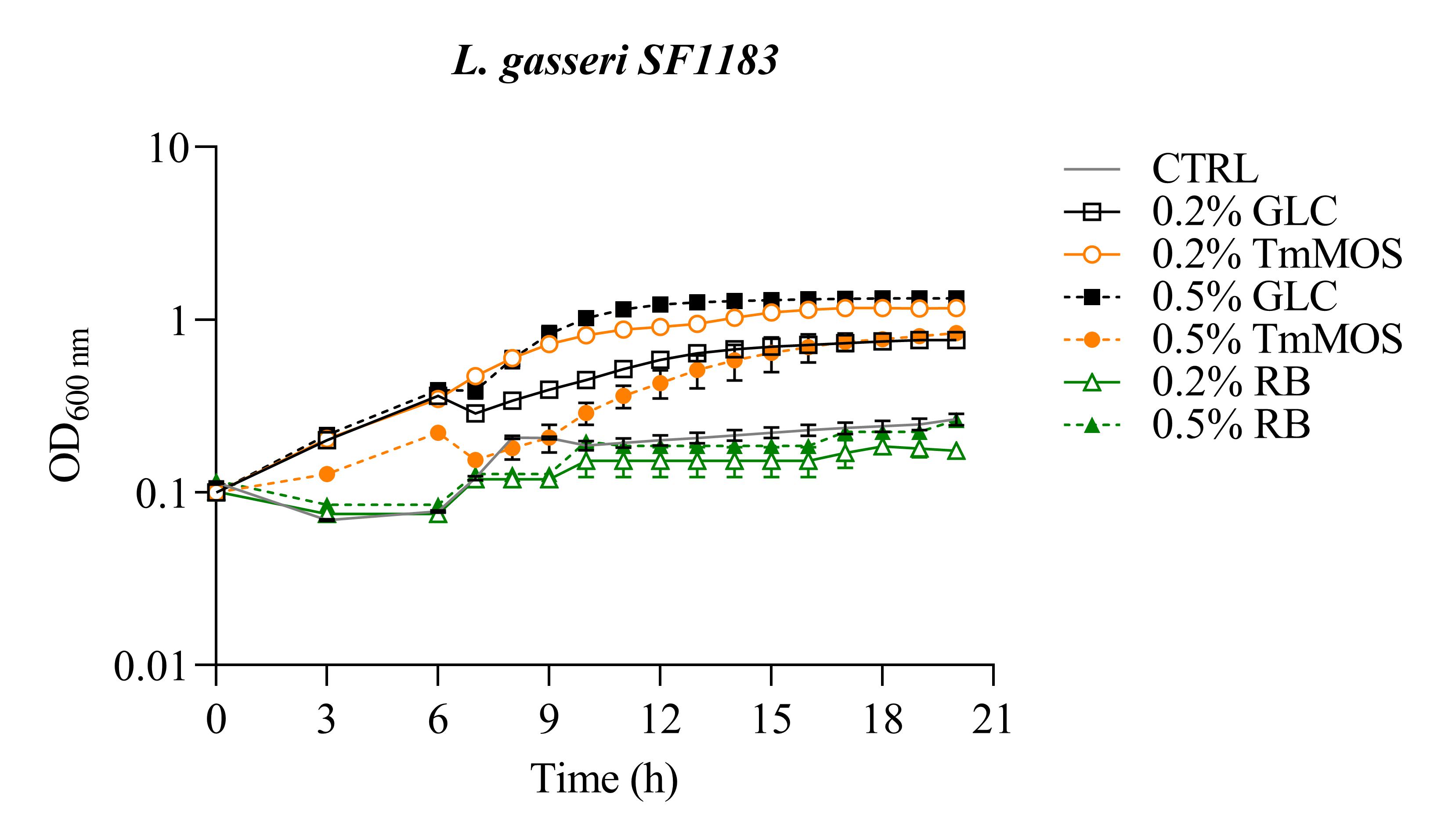


**Figure S5:** Semi-logarithmic plots of growth curves measured as OD 600 of probiotic bacteria tested. Data were expressed as means ± S.D of six replicates of two independent experiments for each condition. CRTL: control, GLC: glucose, RB: reaction blank, TmMOS: reaction product of SCGs MW AK1 140 °C hydrolysis by TmManB5.
